# Comparative transcriptomic profiling of drug-metabolizing enzymes and drug transporters in rabbit ocular subtissues, liver, and duodenum

**DOI:** 10.1016/j.dmd.2025.100099

**Published:** 2025-05-20

**Authors:** Mengyue Li, Brian J. Sanderson, Jennifer Leigh Hackett, Sayuri Sammanani Niyangoda, Huan Gong, Michael A. Johnson, Michael Zhuo Wang

**Affiliations:** 1 Department of Pharmaceutical Chemistry, School of Pharmacy, University of Kansas, Lawrence, Kansas, USA; 2 Department of Molecular Biosciences, University of Kansas, Lawrence, Kansas, USA; 3 Genome Sequencing Core, University of Kansas, Lawrence, Kansas, USA; 4 Department of Chemistry, College of Liberal Arts & Sciences, University of Kansas, Lawrence, Kansas, USA

**Keywords:** Transcriptomics, RNA-seq, Ocular, Drug metabolism, Drug transporter

## Abstract

The increasing prevalence of diabetes and the aging population have led to a growing market for ophthalmic pharmaceutical drugs. However, the development of ophthalmic drugs is hampered by the poor understanding of drug-metabolizing enzymes (DMEs) and drug transporters (DTs) in complex ocular tissues. The rabbit is the most commonly used preclinical model for human ocular diseases. The purpose of this study was to compare relative gene expression of DMEs and DTs in various rabbit ocular subtissues, including cornea, iris–ciliary body, vitreous humor, and retina–choroid complex. Rabbit liver and duodenum samples were also included to investigate gene expression differences among the eye, liver, and duodenum. The mRNA transcriptome of the 6 tissue types from 6 rabbits (3 males and 3 females) was sequenced in a single run using RNA-seq for cross-tissue gene expression comparisons. A total of 387 DME and 128 DT genes were identified across the 6 tissues, and distinct expression patterns of DME and DT genes were observed between ocular subtissues vs. liver and duodenum and among different ocular subtissues. Furthermore, specific DMEs and DTs that enriched in each tissue type were identified and relative gene expression of DMEs and DTs across the 6 tissue types was measured. These results are expected to aid ophthalmic drug development by providing a basis for ocular tissue-specific drug disposition/response and informing model-based predictions and interspecies extrapolation.

**Significance Statement:**

This study represents the first comprehensive comparative transcriptomic analysis of drug-metabolizing enzymes and drug transporters in rabbit ocular subtissues, with comparisons to key metabolic organs such as liver and duodenum. Our findings highlight critical tissue-specific differences that have significant implications for ophthalmic drug development and ocular drug delivery systems. The quantitative gene expression levels of drug-metabolizing enzymes and drug transporters in the rabbit eye will aid investigations into ocular drug metabolism and disposition in both preclinical and clinical settings.

## 1 Introduction

Drug metabolism and disposition are crucial in determining therapeutic efficacy and safety during drug discovery and development (Zhang and Tang, 2018). While the liver is the primary site of drug metabolism, extrahepatic tissues such as the eye also play significant roles in localized drug metabolism and clearance. In addition, extrahepatic metabolism could lead to generation of site-specific reactive metabolites and hence organ-specific toxicity (Argikar et al, 2017b). This is particularly relevant given the rising global burden of eye diseases like glaucoma, macular degeneration, and diabetic retinopathy, which will affect an estimated 1.7 billion individuals worldwide by 2050 and can lead to irreversible blindness if left untreated (GBD 2019 Blindness and Vision Impairment Collaborators; Vision Loss Expert Group of the Global Burden of Disease Study, 2021). Consequently, there is an urgent need to develop effective therapies that can prevent or slow the progression of these sight-threatening diseases. However, ophthalmic drug development is challenging due to the complex anatomy and physiology of the eye, including ocular barriers like the cornea and blood–retinal barrier (BRB), which restrict drug penetration into the eye (Gaudana et al, 2010). Furthermore, various ocular tissues contain specialized drug-metabolizing enzymes (DMEs) and drug transporters (DTs) that may significantly influence the local metabolism and disposition of both locally and systemically administered drugs (Nakano et al, 2014).

DMEs play a critical role in the biotransformation of pharmaceutical compounds, converting lipophilic drugs into more hydrophilic metabolites and facilitating their excretion from the body (Almazroo et al, 2017). DMEs are primarily categorized into Phase I and Phase II enzymes. Phase I enzymes include oxidases such as cytochrome P450s (CYPs), reductases such as aldo-keto reductase (AKR), and hydrolases such as carboxylesterases (CES), which are critical for the initial breakdown of many drugs, making them pharmacologically less active or ready for conjugation by phase II enzymes. Phase II enzymes mediate conjugation reactions, often rendering drugs significantly more hydrophilic and ready for excretion (Iyanagi, 2007). DTs are important membrane proteins that facilitate the movement of drugs, metabolites, and other molecules across biological membranes either through ATP-dependent active transport or through ion or concentration gradients (Petzinger and Geyer, 2006). There are 2 major superfamilies of drug transporters, the ATP-binding cassette (ABC) transporters that actively pump drugs in or out of the cells and the solute carrier (SLC) transporters that mediate drug uptake and efflux with their electrochemical gradients or secondary to a coupled transport (Roberts, 2021). In the eye, these transporters regulate drug bioavailability, particularly across the BRB (Vellonen et al, 2018). Understanding the expression profiles of these DMEs and DTs across different ocular tissues is essential for improving ocular drug delivery strategies, minimizing systemic exposure, and enhancing the therapeutic effects of ophthalmic treatments.

Previous studies have reported the expression of DMEs, including CYPs and CESs, and DTs, such as P-glycoprotein (P-gp) and organic anion transporting polypeptides (OATPs) in ocular tissues (Vellonen et al, 2018; Wolf et al, 2022). Recently, several review papers summarized the presence and expression levels of some DMEs and DTs in the ocular tissues of human and several preclinical species (Nakano et al, 2014; Argikar et al, 2017a; Pikuleva, 2023; Hammid and Honkakoski, 2024). However, comprehensive data on the tissue-specific expression patterns of DMEs and DTs in the eye are still limited, especially in the preclinical animal models. Moreover, there is a paucity of comparative data on the expression levels of these DMEs and DTs in ocular tissues relative to key metabolic organs such as liver and duodenum.

To address this knowledge gap, we performed transcriptomic profiling of DMEs and DTs in various rabbit ocular subtissues, including the cornea (C), iris-ciliary body (ICB), vitreous humor (VH), and retina-choroid complex (RC), as well as the liver and duodenum. Rabbits were chosen as the model animal due to the similarity of their ocular physiology to that of humans, particularly in terms of drug absorption and tissue structure (Zernii et al, 2016). This study aims to improve our understanding of the tissue-specific expression patterns of DMEs and DTs in the rabbit eye and study results are expected to aid optimization of drug delivery strategies for ophthalmic therapies, improve interspecies extrapolation between rabbits and humans, and expedite ophthalmic drug development.

## 2 Materials and methods

### 2.1 Chemicals and reagents

Materials used for RNA extraction such as RNaseZap solution, TRIzol reagent, chloroform, ethanol, isopropanol, RNase-free water, 2.8**-**mm ceramic beads, 2-mL reinforced tubes with screw caps and O-rings, Invitrogen RNAlater stabilization solution, phosphate buffered saline (10×), and Mini Bead Mill Homogenizer were purchased from Fisher Scientific. Liquid nitrogen was purchased from the Matheson Tri-Gas Inc. Reagents used for mRNA library preparation such as NEBNext Ultra II Directional RNA Library Prep, NEBNext Poly(A) mRNA Magnetic Isolation Module, and NEBNext Multiplex Oligos for Illumina (96 Unique Dual Index Primer Pairs) were purchased from New England Biolabs.

### 2.2 Rabbit tissue collection

A study protocol (AUS# 210-04) was approved by the Institutional Animal Care & Use Committee at the University of Kansas. New Zealand White rabbits (n = 6, comprising 3 females and 3 males; Charles River Laboratories; weighing 2.0–3.0 kg and aged 8–10 weeks) were individually housed in an environmentally controlled room (12-hour light/dark cycle; temperature 61–72 °F; relative humidity 30%–70%) within the Animal Care Facility of the University of Kansas, and acclimated for at least 2 days after arrival before commencing any research procedures. Rabbits were fed with a commercial pelleted diet and tap water supplied ad libitum. Hay was provided as dietary supplementation. Rabbits were humanely euthanized with a lethal dose of sodium pentobarbital before tissue harvesting. Aqueous humor (AH) was collected transcorneally using a 1-mL syringe fitted with a 25-gauge needle inserted tangentially. The rabbit eyes were enucleated by dissecting the conjunctiva and the optic nerve (leaving approximately a 5–10 mm section of the nerve to prevent loss of intraocular pressure) after deflection of the nictitating membrane. The orbits were rinsed occasionally with saline during the dissection to prevent drying and afterward to remove any adherent tissue or blood (ICCVAM, 2010). Ocular subtissues, including the cornea, ICB, VH, and RC, were carefully dissected from the eyeball immediately after enucleation. In addition, the liver, as well as the initial 30 cm of the small intestine which corresponds to the duodenum (Nath et al, 2016), were subsequently dissected from the same animal. Immediately following the eyeball dissection, the AH and VH were snap-frozen in liquid nitrogen and stored at −80 °C until further analysis. All the other isolated tissues were placed into RNAlater solution and stored at 4 °C overnight. Subsequently, these tissues were removed from the RNAlater solution and stored at –80 °C until further analysis.

### 2.3 RNA extraction and mRNA sequencing

Thawed tissues were homogenized in reinforced tubes containing 2.8-mm ceramic beads and TRIzol Reagent (1 mL/100 mg tissue) using a Mini Bead Mill Homogenizer, and total RNA was extracted according to the TRIzol Reagent instructions. RNaseZap solution was used during the RNA extraction procedure to remove RNase contamination from work surfaces and non-disposable items. Total RNA samples were assessed with Qubit and Agilent TapeStation for quality and quantity. Transcriptome libraries were prepared using the NEBNext Ultra II Directional RNA Library Prep Kit by enriching the mRNA fragments in total RNA samples, converting them to cDNA, and PCR amplifying with unique dual indices to enable multiplexing. These sequencing libraries were combined into a single pool for sequencing and FASTQ data generation on the Illumina NextSeq 2000 instrument at the University of Kansas Genome Sequencing Core.

### 2.4 RNA-seq analysis

**Fig. 1:**
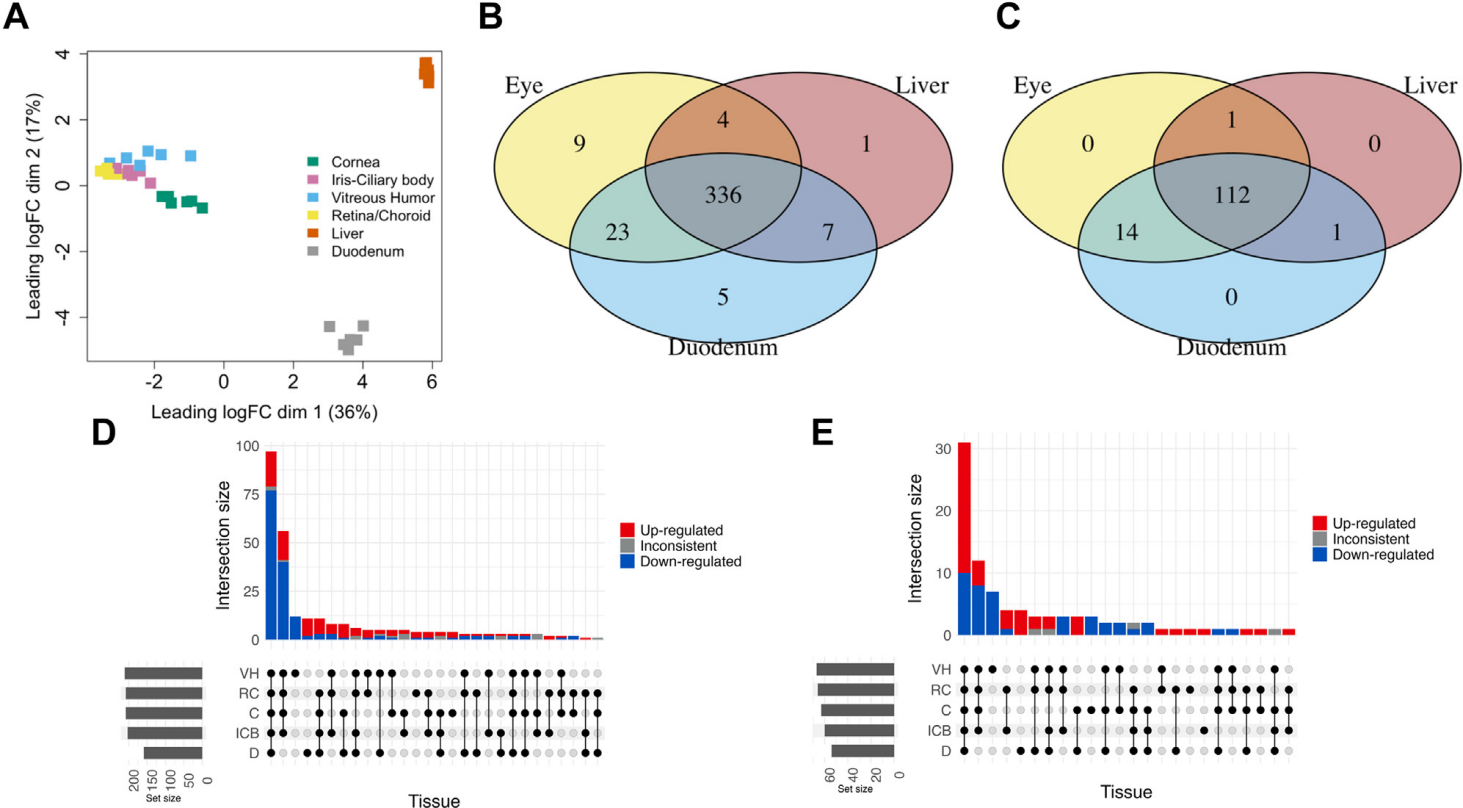
The variability, similarity, and distribution of gene expression profiles among the 6 rabbit tissues and the biological replicates. Multidimensional scaling (MDS) plots were used to assess variability and similarity of gene expression patterns for (A) all expressed genes. Venn diagrams were used to show the overall distribution of (B) DMEs (n = 387) and (C) DTs (n = 128) identified in the rabbit eye, liver, and duodenum. UpSet plots were used to summarize the patterns of differentially expressed (D) DMEs and (E) DTs between all tissues and liver. Bars in the top panels of the UpSet plots are colored based on whether genes were consistently upregulated (red) or downregulated (blue) between the tissues in that set. Bars in the left panel of the UpSet plots represent the total number of genes found to be differentially expressed between the indicated tissues (VH: vitreous humor, RC: retina-choroid complex, C: cornea, ICB: iris-ciliary body, D: duodenum) and the liver. Connected black dots in the bottom panel of the UpSet plots represent common patterns of differential expression between the indicated tissues and the liver.

Raw sequencing reads were processed using the nf-core RNA-seq pipeline version 3.12.0 (Di Tommaso et al, 2017) with Nextflow version 23.04.3 (Ewels et al, 2020). Reads were trimmed for adapter sequences and low-quality bases using Trim Galore version 0.6.7 (Krueger et al, 2021) (Supplemental Table 1), assessed for batch effects using a multidimensional scaling plot (MDS; Fig. 1A). Trimmed reads were aligned to the OryCun 2.0 build of the rabbit genome (GCA_000003625.1) with the Ensembl version 110 annotation using STAR version 2.7.10a (Dobin et al, 2013), and read counts were quantified as both gene raw counts (Supplemental Table 2) and transcripts per million (TPM; Supplemental Table 3) using RSEM version 1.3.1 (Li and Dewey, 2011). The gene raw counts were used to estimate differential gene expression among rabbit cornea, ICB, VH, and RC, duodenum, and liver using DESeq2 version (Love et al, 2014), and log_2_-fold change values were adjusted with the lfcShrink function specifying the apeglm method (Zhu et al, 2019), in R version 4.3.1 (R Core Team, 2023) (Supplemental Table 4). The primary model of differential expression used in this analysis (counts ∼ animal_id + tissue) estimated the main effects of differences between ocular and metabolic tissues, controlling for the differences between animals. To assess the extent to which differences in expression we estimated in this model were driven by differences between male and female rabbits, we ran an additional model (counts ∼ sex + tissue + sex:tissue), which we used to assess the impact of sex on the comparisons of expression between ocular and metabolic tissues.

### 2.5 Candidate gene analysis

A panel of 527 candidate genes for rabbit DMEs (n = 397) and DTs (n = 130) was assembled through manual screening the Ensembl database for pertinent genes (Table 1). The selection of DMEs included Phase I: Cytochrome P450s, Phase I - Oxidases, Phase I - Reductases, Phase I - Hydrolases, and Phase II: transferases. Within each DME category, proteins were grouped into either major or minor subcategory: major DMEs are those previously reported for playing important roles in drug metabolism in humans (Feng et al, 2010; Fukami et al, 2022), and the rest were grouped as minor DMEs. The selection of ABC transporters included the ABCA, ABCB, ABCC, and ABCG subfamilies that were known to be responsible for drug transport in humans, hence grouped as major ABCs, and the rest were grouped as minor ABCs that include those that transport long-chain fatty acid or affect antiviral/antibacterial activities (Roberts, 2021). Similarly, the selection of SLC transporters included OATPs, organic anion transporters (OATs), organic cation transporters (OCTs), and multidrug and toxic compound extrusion transporters (MATEs) that were known to play a major role in transporting clinical drugs, therefore grouped as major SLCs, and the rest were grouped as minor SLCs that were also reported to be involved in drug transport in humans (Roberts, 2021). It should be noted that the major/minor subcategorization was mainly for convenience during analysis and discussion and their significance in drug metabolism and transport can change over time.

**Table 1:** Candidate gene panel for rabbit drug-metabolizing enzymes and drug transporters

Candidate Gene Panel of Rabbit DMEs and DTs (n = 527)				Numbers
Phase I Metabolizing enzymes (n = 294)	Oxidases (n = 134)	Cytochrome P450s	Cytochrome P450s	41
		Non-CYP450 oxidases (major)	Flavin-containing monooxygenases (FMO)	5
			Monoamine oxidases (MAO)	2
			Aldehyde oxidase (AO), xanthine dehydrogenase (XHD)	5
			Alcohol and Aldehyde dehydrogenases (ADH and ALDH)	17
		Non-CYP450 oxidases (minor)	Peroxidases, NADPH oxidase, etc.	64
	Reductases (n = 55)	Major reductases	Aldo-keto reductase (AKR)	8
			Carbonyl reductase (CBR)	3
			Dehydrogenase/reductase (DHR)	9
			Short chain dehydrogenase/reductase (SDR)	5
			NAD(P)H quinone dehydrogenase (NQO)	2
			Cytochrome p450 oxidoreductase (POR)	1
		Other reductases	Thioredoxin reductase, prostaglandin reductase, etc	27
	Hydrolases (n = 105)	Major hydrolases	Carboxylesterases (CES)	5
			Arylacetamide deacetylase (AADAC)	2
			Paraoxonases (PON)	3
			*α*/*β* hydrolase domain (ABHD)	13
			Epoxide hydrolase (EPHX)	3
			Fatty acid amide hydrolase (FAAH)	2
			Butyrylcholinesterase (BCHE) and esterase D (ESD)	2
		Other hydrolases	Carboxypeptidase, albumin, etc.	75
Phase II Metabolism enzymes (n = 103)	Transferases (n = 103)	Major transferases	UDP-glucuronosyltransferases (UGT)	8
			Glutathione-S-transferases (GST)	10
			Sulfotransferases (SULT)	10
			N-acetyltransferases (NAT)	2
		Other transferases	Methyltransferases, amino acid acyl transferases, etc	73
Transporters (n = 130)	ATP-binding cassette (ABC) family (n = 40)	Major ABCs	ABC subfamily A (ABCA)	7
			ABC subfamily B (ABCB)	10
			ABC subfamily C (ABCC)	11
			ABC subfamily G (ABCG)	4
		Other ABCs	ABC subfamily D, subfamily E, and subfamily F	8
	Solute carrier (SLC) family (n = 90)	Major SLCs	Solute carrier organic anion transporter family (OATPs)	9
			SLC22: Organic anion transporters and organic cation transporters (OATs and OCTs)	10
			SLC47: multidrug and toxic compound extrusion (MATE)	2
		Other SCLs	SLC drug-transporting families (SLC1, 3, 6, 7, 10, 15, 28, 29, 35)	69

For each category of DMEs and DTs, 3 types of analyses were performed. First, differential expression profiles of major DMEs or DTs across the 6 rabbit tissues were visualized via heatmaps using log_2_-transformed, DESeq2-normalized gene expression levels (Love et al, 2014; Rosati et al, 2024) and with hierarchical clustering using the R package dendextend v1.17.1 (Galili, 2015). Second, the log_2_ transformed TPM values were used to rank the expression of all DME and DT genes within each tissue group (Zhao et al, 2021b) and the top 10 expressed DME or DT genes within each tissue group were visualized using bar plots (GraphPad Prism 10.0; GraphPad Software). Third, DMEs or DTs from major subcategories with significant ocular tissue expression were selected as “ocular” DMEs or DTs and their gene expression levels across the 6 tissue groups were quantified, compared, and visualized using box-and-whisker plots showing the center line as the median, the box as the 25th and 75th percentiles, and the whiskers as the min and max values (GraphPad Prism 10.0). The Benjamini-Hochberg adjusted *P*-values generated from the DESeq2 analysis were employed assess the significance of differences in gene expression between the ocular subtissues and duodenum compared with the liver, adjusted *P*-values were denoted by ns (not significant) for *P* ≥ .01, ∗ for 0.01>*P* ≥ .001, ∗∗ for .001 > *P* ≥ .0001, and ∗∗∗ for *P* < .0001. Ocular DMEs or DTs were searched in the Human Eye Transcriptome Atlas (Wolf et al, 2022) (Version 3.0; access date: April 10, 2025) to explore their expression in human ocular subtissues and contextualize the results of the present work. Finally, UpSet plots were created for the DME and DT genes to summarize the patterns of differential expression between all tissues and liver (adjusted *P* value < .01, | log_2_ fold-change | > 2), using the R package ComplexUpset v1.3.5 (Lex et al, 2014; Krassowski et al, 2020).

## 3 Results

### 3.1 Data quality and initial assessment

RNA Integrity Number (RIN) values for all tissue RNA samples (a total of 36) were between 8.9 to 10.0 except for 4 VH samples (out of the 6 VH samples), which had RIN values around 6. This may be due to the difficulty of extracting mRNA from the gelatinous vitreous humor using the TRIzol reagent and/or the low mRNA expression in the VH. We note that the VH libraries resulted in an order of magnitude fewer reads than the other libraries and thus have a proportionally smaller total gene counts across the transcriptome (Supplemental Table 1). Overall, our RNA-seq libraries generated an average of 35,299,422 ± 14,728,956 single-end reads (mean ± SD), of which an average of 48% mapped uniquely to the reference transcriptome (Supplemental Table 1). The mean quality score of all sequence reads was over 30, confirming that the reads were of good quality (data not shown). A total of 22,457 genes were identified based on the inclusion criteria that a gene must be expressed (non-zero count) in more than one sample to be included in the filtered dataset, which helps to reduce the influence of noise or technical artifacts from low-count genes that might only appear in a single sample by chance (Love et al, 2015). The variability and similarity of the gene expression profiles among the 6 rabbit tissues and the biological replicates were shown using the MDS plots for all identified genes (Fig. 1A). First, all biological replicates of each tissue type clustered together and each ocular subtissue, ie, cornea, ICB, VH, and RC, occupied a distinct space in the plot, suggesting good reproducibility and successful dissection of the respective subtissue. Second, ocular subtissues were well delineated from the liver and duodenum, indicating large differences in the gene expression profiles between ocular tissues and other key organs of drug disposition. Interestingly, ICB and RC resembled each other more than cornea and VH did. The complete list of candidate genes for rabbit DMEs and DTs included in this study was included in Supplemental Table 5. Out of the 527 candidate DME and DT genes, 387 (out of 397) DME and 128 (out of 130) DT genes were identified in the rabbit ocular, liver, and/or duodenum tissues. The overall distribution and overlap of these identified DMEs and DTs in the rabbit eye, liver, and duodenum were visualized in the Venn diagrams (Fig. 1, B and C). 336 (out of 387) DME and 112 (out of 117) DT genes were commonly shared by the rabbit ocular, liver, and duodenum tissues, which highlights that the majority of DMEs and DTs were commonly expressed across the eye, liver, and duodenum, indicating a shared baseline metabolic capability. Candidate genes that are differentially expressed (adjusted *P* value < .01, | log_2_ fold-change | > 2) in the ocular subtissues and duodenum compared to the liver were visualized by the UpSet plots to illustrate their distribution across these tissues. The analysis reveals that most differentially expressed DME genes in the ocular subtissues and/or duodenum were downregulated relative to the liver. In contrast, most of the differentially expressed DT genes in the ocular subtissues and/or duodenum were upregulated compared to the liver. These findings are consistent with the liver’s primary role as a metabolic organ and the eye and duodenum’s functions as biological barriers.

### 3.2 Phase I - Cytochrome P450s

**Fig. 2:**
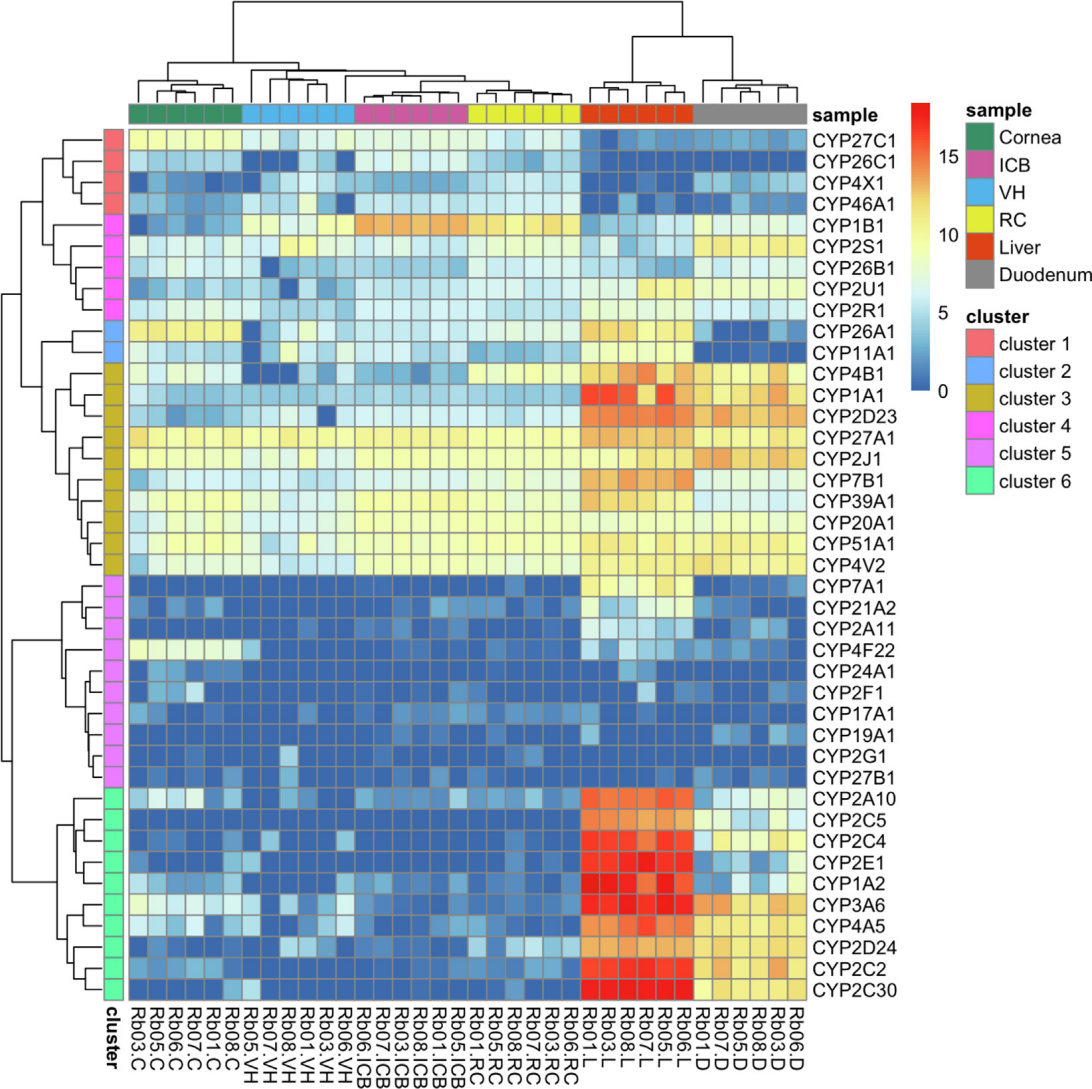
The mRNA gene expression heatmap of cytochrome P450 genes (n = 41) in rabbit ocular subtissues, the liver, and the duodenum. The log_2_-transformed DESeq2 normalized counts were used to generate the heatmap and results were clustered as described in Materials and Methods.

The expression pattern of the 41 candidate rabbit CYP enzymes across the 6 tissue groups was visualized and clustered using a heatmap of the DESeq2-normalized, log_2_-transformed mRNA expression (Fig. 2). Tissue-specific expression patterns were evident with liver exhibiting the highest overall expression of most CYP enzymes, followed by duodenum and then various ocular subtissues. For instance, cluster 6 consisted of CYPs belonging to the CYP1, 2, 3, and 4 subfamilies, such as CYP1A2, CYP2Cs, CYP2E1, CYP3A6, and CYP4A5, which showed the highest expression in the liver, followed by the duodenum, with minimal expression in the ocular tissues. The human orthologs of these CYP enzymes (eg, CYP1A2, CYP2C9, CYP2C19, CYP2D6, CYP2E1, and CYP3A4) are known to play major roles in drug metabolism (Zanger and Schwab, 2013; Zhao et al, 2021a). CYPs in clusters 1 to 4 (eg, CYP2J1, CYP4V2, CYP20A1, CYP26A1, CYP27A1, CYP27C1, CYP39A1, and CYP51A1) showed notable expression in various ocular subtissues, indicating their essential roles in the eye. These CYP enzymes are involved in the metabolism of endogenous compounds such as sterols, vitamin A, and fatty acids (Manikandan and Nagini, 2018). For example, CYP51A1 is the lanosterol 14*α*-demethylase and plays an important role in the sterol and cholesterol biosynthesis (Debose-Boyd, 2007). Notably, several CYPs exhibited the highest expression in ocular subtissues. For example, CYP1B1 exhibited high expression, particularly in the ICB and RC, aligning with its known role in steroid metabolism and its association with ocular diseases such as glaucoma (Vasiliou and Gonzalez, 2008). CYPs in cluster 5 generally had low expression in all examined tissues, but a few exceptions stood out, eg, CYP4F22 and CYP7A1, which had moderate expression in the cornea and liver, respectively.

**Fig. 3:**
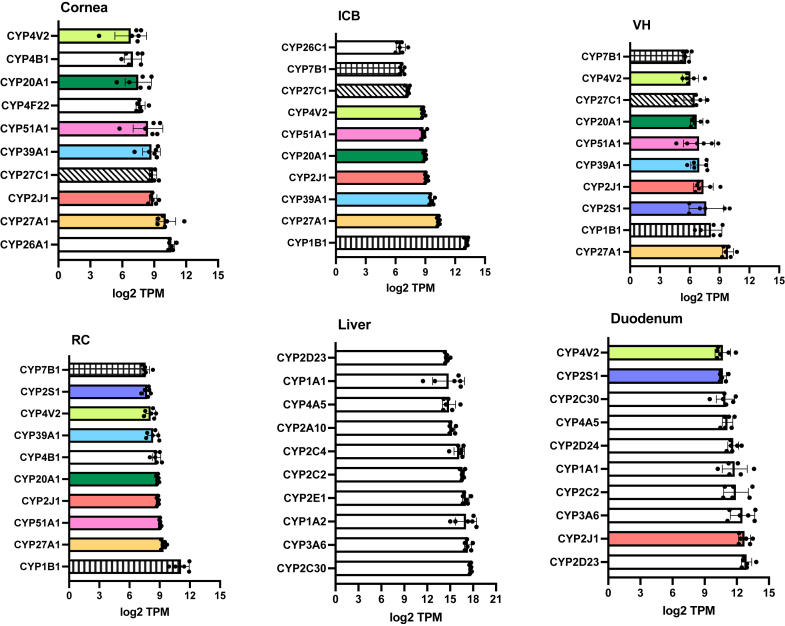
Top 10 most abundantly expressed cytochrome P450 genes in the rabbit cornea, ICB, VH, RC, liver, and duodenum. The log_2_-transformed transcripts per million (TPM) values were used to rank expression within each tissue type. Genes identified in the top 10 lists of multiple tissue types were color/pattern-coded to facilitate visual identification.

The expression level of DMEs and DTs within each tissue was ranked using the averaged log_2_ transformed TPM values, which were provided in Supplemental Table 6, and the top 10 expressed CYP enzymes were visualized in the bar plots (Fig. 3). The results highlight 6 commonly expressed CYPs in all ocular subtissues, including CYP2J1, CYP4V2, CYP20A1, CYP27A1, CYP39A1, and CYP51A1. Three additional CYPs (ie, CYP1B1, CYP7B1, CYP27C1) were among the top 10 expressed CYP enzymes in 3 out of the 4 ocular subtissues. Two other CYPs (ie, CYP2S1 and CYP4B1) are among the top 10 expressed CYP enzymes in 2 out of the 4 ocular subtissues. Three more CYPs (ie, CYP4F22, CYP26A1, and CYP26C1) are among the top 10 expressed CYP enzymes in only one out of the 4 ocular subtissues. Interestingly, CYP2S1 is expressed in the duodenum and the posterior part of the eye (VH and RC). As expected, none of the top 10 expressed liver CYPs is found in the top 10 expressed ocular CYPs and only 3 of the top 10 expressed duodenum CYPs are found in the top 10 expressed ocular CYPs. These data provide a comprehensive overview of the tissue-specific CYP expression, which may reflect their specialized metabolic roles in the eye, liver, and duodenum.

**Fig. 4:**
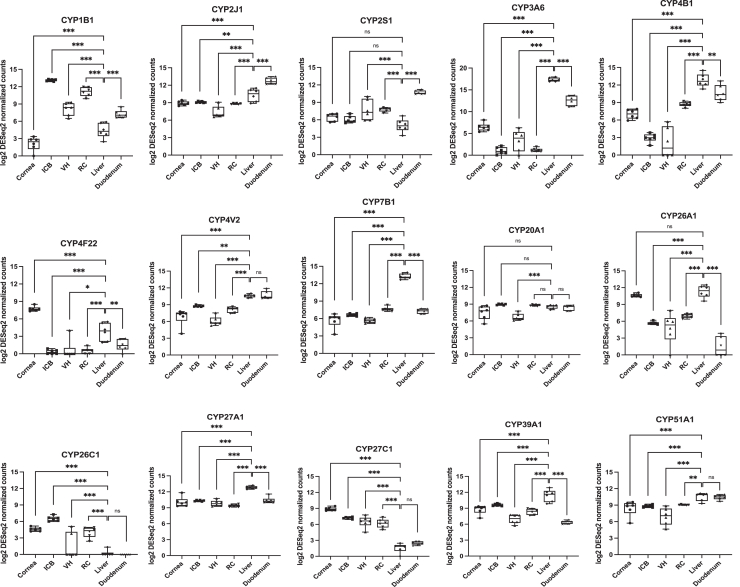
The relative expression levels of ocular cytochrome P450 genes in the rabbit cornea, ICB, VH, RC, liver, and duodenum. The log_2_-transformed DESeq2 normalized counts were used to generate box-and-whisker plots, and the Benjamini-Hochberg adjusted *P*-values from DESeq2 were employed to compare the gene expression levels in the ocular subtissues and duodenum to the liver, as described in the Materials and Methods.

Furthermore, 15 CYP enzymes that generally exhibited moderate to high expression in the eye (namely, ocular CYPs) were selected for quantitative comparative analysis using box-and-whisker plots (Fig. 4). These ocular CYPs include CYP1B1, CYP2J1, CYP2S1, CYP3A6, CYP4B1, CYP4F22, CYP4V2, CYP7B1, CYP20A1, CYP26A1, CYP26C1, CYP27A1, CYP27C1, CYP39A1, and CYP51A1. Among these, CYP26C1 and CYP27C1 were the most ocular-specific CYPs, showing higher expression in all ocular subtissues than the liver and duodenum. CYP1B1 showed higher expression level in the ICB, VH, and RC, compared to the liver; CYP2S1 expression in the eye shows similar or higher levels than the liver, with highest expression level in the duodenum; CYP4F22 showed exclusively higher expression in the cornea; CYP26A1 exhibited similarly high expression in the cornea and liver; CYP20A1 showed similar expression across all the tissues, with the lowest in the VH. The rest of ocular CYPs all showed considerable expression in the ocular subtissues, but their highest expression was observed either in the liver or duodenum. This differential expression analysis highlights the tissue-selective expression of CYP enzymes and ocular CYPs may play an important role in the local drug metabolism in the eye.

### 3.3 Phase I – Hydrolases

The major categories of phase I hydrolases involved in human drug hydrolysis include CESs, arylacetamide deacetylase (AADAC), paraoxonases (PONs), *α*/*β* hydrolase domains (ABHDs), epoxide hydrolases (EPHXs), fatty acid amide hydrolases (FAAHs), butyrylcholinesterase (BCHE) and esterase D (ESD). In this study, 29 rabbit hydrolase genes belonging to the aforementioned major hydrolase categories were analyzed to compare their expression patterns across 6 tissue groups, and the results were visualized using a heatmap (Supplemental Fig. 1). Although the liver exhibiting the highest overall hydrolase expression, most of the hydrolases were also highly expressed across all tissues. Hydrolases such as CES1, CES2, CES3, EPHX1, AADAC, PON1 and PON3 were highly expressed in the liver, consistent with their significant roles in drug metabolism (Fukami et al, 2022). Among them, CES1, AADAC, and PON3 also exhibited high expression in the cornea, while EPHX1 showed high expression across all tissues. Additionally, hydrolases like FAAH, PON2, ESD, and 10 ABHDs showed notable expression across all tissues, suggesting their essential functions in the eye, liver, and duodenum. For instance, ESD, a serine hydrolase involved in detoxification of formaldehyde, was reported to be expressed in multiple human tissues at the RNA level (Karlsson et al, 2021). In contrast, BCHE exhibited similar expression levels between ocular tissues and the duodenum, with the lowest expression level observed in the liver.

A total of 105 candidate hydrolases (including 59 amidases/peptidases) were included in this study, and the top 10 expressed hydrolases within each tissue were identified using the averaged log_2_-transformed TPM values and visualized in the bar plots (Supplemental Fig. 2). The results highlighted the high abundance of peptidases across all ocular subtissues, with 6 of the top 10 being peptidases within each tissue type. Such peptidases include aminopeptidase-like 1 (NPEPL1), aspartyl aminopeptidase (DNPEP), carboxypeptidase Q (CPQ), carboxypeptidase X1 (CPXM1), CPXM2, leucine aminopeptidase 3 (LAP3), matrix metallopeptidase 2 (MMP2), methionyl aminopeptidase 2 (METAP2), N-terminal asparagine amidase (NTAN1), puromycin-sensitive amino peptidases (NPEPPS), and serine carboxypeptidase 1 (SCPEP1). In addition to peptidases, EPHX1 and ESD were highly expressed across all ocular subtissues. ABHD4 was highly expressed in the ICB, VH, and RC. CES1 showed high expression in the cornea and ICB, while nitrilase 1 (NIT1) and PON2 was highly expressed in the VH and RC, respectively. In contrast, the top 10 expressed hydrolases in the liver are ranked as follows: albumin (ALB), EPHX1, CES1, PON1, PON3, carboxypeptidase B2 (CPB2), glucuronidase beta (GUSB), CES2, carboxypeptidase N subunit 1 (CPN1), and AADAC, while the top 10 expressed hydrolases in the duodenum are ranked as follows: pancreatic lipase (PNLIP), carboxyl ester lipase (CEL), carboxypeptidase A1 (CPA1), EPHX1, CPB1, CPA2, PON3, CES2, LAP3, and alanyl aminopeptidase (ANPEP). Interestingly, most of the hydrolases highly expressed in duodenum tissues were also peptidases, like ocular subtissues. These findings provide a comprehensive overview of tissue-specific hydrolase expression, reflecting their specialized metabolic roles in the eye, liver, and duodenum.

Furthermore, 9 major hydrolases (AADAC, ABHD4, BCHE, CES1, EPHX1, ESD, FAAH, PON2, PON3) and 11 peptidases (CPQ, CPXM1, CPXM2, DNPEP, LAP3, METAP2, MMP2, NPEPL1, NPEPPS, NTAN1 and SCPEP1) that generally exhibited high expression in the eye (namely, ocular hydrolases) were selected for quantitative comparative analysis using box-and-whisker plots (Supplemental Fig. 3). Among the major hydrolases, compared to the expression levels in the liver, BCHE exhibited higher expression level in all the ocular subtissues and duodenum; ABHD4, CES1, EPHX1, ESD, FAAH, and PON2 all showed similar or less but appreciable expression levels in various ocular subtissues; AADAC and PON3 exhibited much lower expression level (<1%) compared to the liver. However, peptidases CPXM1, CPXM2, and MMP2 exhibited higher expression level in all the ocular subtissues compared to the liver; DNPEP, METAP2, and NTAN1 all exhibited similar expression levels in all the ocular subtissues compared to the liver, whereas peptidases CPQ, LAP3, NPEPL1, NPEPPS and SCPEP1 all exhibited similar or slightly lower expression levels in various ocular tissues compared to the liver. This differential expression analysis highlights the high abundance of certain hydrolases, eg, CES1 and EPHX, and peptidases, eg, carboxypeptidases and MMP2, in the ocular subtissues.

### 3.4 Phase I - Non-CYP oxidases

The major phase I non-CYP oxidases involved in human drug hydrolysis include flavin-containing monooxygenase (FMOs), monoamine oxidases (MAOs), aldehyde oxidases (AOXs), xanthine dehydrogenase (XDH), alcohol dehydrogenases (ADHs), and aldehyde dehydrogenases (ALDHs) (Feng et al, 2010; Fukami et al, 2022). In this study, 29 major rabbit non-CYP oxidases belonging to the aforementioned major non-CYP oxidase categories were analyzed to compare their expression patterns across the 6 tissue groups, and the results were visualized using a heatmap (Supplemental Fig. 4). Tissue-specific expression patterns were observed, with the liver, again, exhibiting the highest overall oxidase expression, followed by the duodenum, and then various ocular subtissues. Notably, ALDH1A1 showed very high expression level in the liver, cornea, and duodenum, and moderate level in the ICB, VH, and RC. Additionally, ADH5, ALDH2, ALDH6A1, ALDH7A1, AOX1, and MAOB also showed considerable expression levels in the various ocular subtissues, albeit less than in the liver. XDH exhibited the highest expression level in the cornea, and AOX4 was exclusively expressed in the cornea samples. ALDH1A2, ALDH1A3, ALDH1L2, ALDH18A1, AOX3, and MAOA had higher expression levels in most of the ocular subtissues than in the liver.

A total of 93 candidate non-CYP oxidases were included in this study, the top 10 expressed non-CYP oxidases within each tissue were identified using the averaged log_2_-transformed TPM values and visualized in the bar plots (Supplemental Fig. 5). The results highlighted the commonly high abundant non-CYP oxidases across all the ocular subtissues, including ADH5, ALDH1A1, ALDH7A1, glyceraldehyde-3-phosphate dehydrogenase (GAPDH), glutathione peroxidase 1 (GPX1), and oxoglutarate dehydrogenase (OGDH). Additionally, ALDH2, glyceraldehyde-3-phosphate dehydrogenase spermatogenic (GAPDHS), GPX3, and lysyl oxidase-like 1 (LOXL1) were highly abundant in most of the ocular subtissues. Among these, ALDH1A1, ALDH2, and GPX1 were also highly expressed in the liver, and OGDH, GPX1, GAPDH, ALDH2, ALDH1A1 were high in the duodenum.

Furthermore, 14 non-CYP oxidases that generally exhibited moderate to high expression in the eye and belonged to the major phase I non-CYP oxidases (namely, ocular non-CYP oxidases) were selected for quantitative comparative analysis using box-and-whisker plots (Supplemental Fig. 6). These ocular non-CYP oxidases including ADH5, ALDH1A1, ALDH1A2, ALDH1A3, ALDH1L2, ALDH2, ALDH6A1, ALDH7A1, ALDH18A1, AOX1, AOX3, MAOA, MAOB, and XDH. Among these, AOX3 and ALDH18A1 were the most ocular-specific non-CYP oxidases, showing higher expression in all ocular subtissues than the liver and duodenum; ALDH1A2, ALDH1A3, ALDH1L2, and MAOA showed higher expression level in most of the ocular subtissues than the liver; XDH exhibited the highest expression level in the cornea; whereas the remaining enzymes showed considerable expression in the ocular subtissues, but their highest expression was observed in the liver.

### 3.5 Phase I **-** Reductases

The major phase I reductases involved in human drug metabolism include AKRs, carbonyl reductases (CBRs), dehydrogenase/reductases (DHRs), short chain dehydrogenase/reductases (SDRs), NAD(P)H quinone dehydrogenase 1 (NQO1), and cytochrome P450 oxidoreductase (POR) (Feng et al, 2010; Fukami et al, 2022). In this study, 28 rabbit reductases belonging to the aforementioned major reductases categories were analyzed to compare their expression patterns across 6 tissue groups, and the results were visualized using a heatmap (Supplemental Fig. 7). Most reductases demonstrated ubiquitous expression across all 6 tissues, with the liver having the highest overall expression, followed by the duodenum, and then the ocular subtissues, such reductases including AKR1A1, AKR7A3, AKR7L, CBR1, DHRS1, DHRS4, DHRS7, DHRS11, NQO2, and POR. In contrast, AKR1B10 showed the highest expression level in duodenum, followed by various ocular subtissues, then the liver. AKR1B1 showed the highest expression level in the VH, moderate level in the other ocular subtissues and the duodenum, minimal expression in the liver. Additionally, CBR3, NQO1, SDR39U1, DHRS7B, SDR16C5 and DHRS7C showed moderate expression level in all ocular subtissues, which also seemed to be higher than their levels in the liver or duodenum.

In total, 55 candidate reductases were included in this study, and the top 10 expressed reductases within each tissue were identified using the averaged log_2_-transformed TPM values and visualized in the bar plots (Supplemental Fig. 8). Several reductases were highly expressed across all ocular subtissues, including AKR1A1, AKR1B10, cytochrome b (CYTB), DHRS1, methionine sulfoxide reductase B1 (MSRB1), and quinoid dihydropteridine reductase (QDPR). In addition to these commonly high abundance reductases, POR was highly expressed in the cornea, ICB, and RC; AKR1B1 was abundant in cornea and VH; cytochrome b5 reductase 3 (CYB5R3) was high in cornea and ICB; pyrroline-5-carboxylate reductase 2 (PYCR2) was highly expressed in VH and RC; CBR3 was abundant in cornea; metalloreductase STEAP4 was abundant in the ICB, AKR7L and CBR1 were abundant in VH, fatty acyl-CoA reductase 1 (FAR1) and cytochrome b reductase 1 (CYBRD1) were abundant in RC. Among these top-expressed ocular reductases, AKR1A1, CYTB, CBR1, DHRS1, POR, and QDPR were also highly expressed in the liver, and AKR1A1, AKR1B10, AKR7L, CYBRD1, CYTB, and DHRS1 were high in the duodenum.

Furthermore, 10 reductases that generally exhibited moderate to high expression in the eye and belonged to the major phase I reductases (namely, ocular reductases) were selected for quantitative comparative analysis using box-and-whisker plots (Supplemental Fig. 9). These ocular reductases included: AKR1A1, AKR1B1, AKR1B10, AKR7L, CBR1, CBR3, DHRS1, NQO1, POR, and SDR16C5. Among these, SDR16C5 was the most ocular-specific reductase, showing higher expression levels in all the ocular subtissues than in the liver and duodenum; AKR1B1 and AKR1B10 showed higher expression levels in all the ocular subtissues than in the liver; CBR3 had the highest expression level in the cornea and lowest expression level in the RC, with similar expression level across ICB, VH, and liver; the other reductases all showed considerable expression in the ocular subtissues, but their highest expression was observed in the liver.

### 3.6 Phase II **-** Transferases

The major phase II transferases involved in human drug conjugation include UDP-glucuronosyltransferase (UGT), glutathione S-transferase (GST), sulfotransferase (SULT), and N-acetyltransferases (NATs) (Jancova et al, 2010; Almazroo et al, 2017). In this study, 25 rabbit transferases belonging to these 4 major transferase families were analyzed to compare their expression patterns across 6 tissue groups, and the results were visualized using a heatmap (Supplemental Fig. 10). Overall, the liver showed the highest expression of most of the transferases, followed by the duodenum, and then various ocular subtissues. For example, GSTA5, GSTM1, GSTM2, GSTO1, SULT1A1, GSTZ1, and SULT1C2 were expressed in all 6 tissues, with the highest levels observed in the liver, followed by the duodenum, and lower levels in the ocular subtissues. In contrast, GSTM3, GSTA4, SULT4A1, and NAT10 were more highly expressed in the ocular subtissues compared to the liver and duodenum. Additionally, UGT2s, SULT2s, and SULT3s were largely restricted to the liver and/or duodenum, whereas UGT1A6 and UGT8 could also be detected in the cornea and RC, respectively.

A total of 103 candidate transferases were included in this study, and the top 10 expressed transferases in each tissue were identified using the averaged log_2_-transformed TPM values (Supplemental Fig. 11). The results highlighted the common top-expressed transferases across all ocular subtissues, most of which were GSTs, microsomal GSTs (MGSTs), N(alpha)-acetyltransferases (NAAs), and N-acetyltransferase domain-containing protein (G1TY16). For example, among the top 10 expressed transferases within each tissue group, GSTA5, GSTM2, GSTM3, G1TY16, MGST3, and NAA20 were consistently highly ranked across all ocular subtissues, with GSTA5, GSTM2, GSTM3, G1TY16, and MGST3 also ranked to the top 10 transferase in the duodenum. Catechol-O-methyltransferase (COMT), and indolethylamine N-methyltransferase (INMT) were also highly expressed in many of the ocular subtissues. Eight out of 10 top transferases in the duodenum were GSTs and MGSTs. In contrast, the liver had high levels of UGT1A6, SULTs, and GSTs.

Nine transferases that showed moderate to high expression in the eye and belonged to the major phase II transferases (namely, ocular transferases) were selected for quantitative comparative analysis using box-and-whisker plots (Supplemental Fig. 12). These ocular transferases included GSTA4, GSTA5, GSTM2, GSTM3, GSTZ1, NAT10, SULT1A1, SULT4A1, and UGT1A6. Among these ocular transferases, GSTM3 showed higher expression level in all the ocular subtissues and duodenum compared to the liver; SULT4A1 exhibited the highest expression level in the VH and RC, and similarly moderate expression levels in the other tissues; GSTA4 and NAT10 had higher or similar expression levels in the ocular subtissues compared to the liver and duodenum; the other transferases all showed considerable expression in the ocular subtissues, but their highest expression was observed in the liver.

### 3.7 ABC transporters

The ABCA, ABCB, ABCC, and ABCG subfamilies have been reported to be involved in the drug transport (Roberts, 2021) and were defined as major ABCs in Table 1. There are 29 such major ABCs were identified as expressed in the rabbit tissues, and hence were included to analyze their expression patterns across 6 tissue groups using a heatmap (Supplemental Figure 13). Most of the ABC transporters were ubiquitously expressed in all the 6 tissue groups, several of them showed exclusive expression in certain tissues. For example, ABCB11, ABCC11, ABCG5, and ABCG8 seemed to display restricted expression in the ocular subtissues, being mainly localized to the liver and duodenum. In contrast, ABCA4, ABCB9, and ABCG4 were predominantly expressed in the ocular subtissues, with little to no expression levels in the liver and the duodenum. ABCA12 and ABCC12 had low expression in all 6 tissues. ABCA6, ABCB1, ABCC2, ABCC3, and ABCC6 showed the highest expression level in the liver or duodenum, and moderate expression level in the ocular subtissues. The remaining major ABC transporters were ubiquitously expressed across all tissues, showing comparable expression levels.

In addition to the major ABCs, there are 11 other ABC transporters from the ABCD, ABCE, and ABCF subfamily. In total, 40 candidate ABC transporters were analyzed to identify the top 10 expressed within each tissue, using averaged log_2_-transformed TPM values, which were visualized in bar plots (Supplemental Fig. 14). The results highlighted several top-expressed ABC transporters across all ocular subtissues, ie, ABCE1, ABCF1, ABCF2, and ABCF3. Among them, ABCE1, ABCF1, and ABCF3 were also ranked in the top 10 list for the duodenum, but none in the top 10 list for the liver. Although these transporters were not known for drug transport, their peculiar expression patterns in the ocular subtissues and duodenum indicate a potentially important role for these transporters in the eye and duodenum. Among the drug-transporting major ABCs, ABCB3, ABCB6, ABCB7, ABCB8, and ABCC4 were the highest expressed ABCs in the cornea. In the ICB, ABCA3, ABCB1, ABCC4, ABCG2, and ABCG4 were the most prominent. In the VH, ABCA3, ABCA4, ABCB8, and ABCG4 showed the highest expression, while in the RC, ABCA4, ABCA8, ABCG2, and ABCG4 were the most expressed.

Furthermore, 15 ABC transporters with key roles in human drug transport and high expression in ocular subtissues (namely, ocular ABC transporters) were selected for further quantitative comparative analysis using box-and-whisker plots (Supplemental Fig. 15). These include ABCA1(cholesterol efflux regulatory protein, CERP), ABCA3, ABCA4 (retinal-specific ABC, ABCR), ABCA8, ABCB1 (P-gp), ABCB3 (antigen peptide transporter 2, TAP2), ABCB6 (mitochondrial ABC transporter 3, MTABC3), ABCB7, ABCB8 (mitochondrial ABC protein, MABC1), ABCB9 (TAP-like ABC transporter, TAPL), ABCC1 (multidrug resistance-associated protein 1, MRP1), ABCC4 (MRP4), ABCC5 (MRP5), ABCG2 (breast cancer resistance protein, BCRP), and ABCG4. Among them, ABCR, TAPL, and ABCG4 were the most ocular-specific efflux transporters, showing higher expression levels in all the ocular subtissues than in the liver and duodenum; ABCB8, MRP1, and MRP4 exhibiting higher or similar expression levels in the ocular subtissues than in the liver; MRP5 showed the highest expression level in the RC, and lowest in the cornea, with similar expression level across the ICB, VH, liver, and duodenum. BCRP had the highest expression level in the ICB, with RC, liver, and duodenum also showed similarly high expression levels. The other ABC transporters all showed considerable expression levels in all the ocular subtissues, at a level either similar to or lower than the levels in the liver.

### 3.8 SLC transporters

The SLCO family (OATPs), the SLC22 family (OATs and OCTs), and the SLC47 family (MATEs) are major categories of SLC transporters that play important role in the human drug transport. There are 20 of them that were identified in the rabbit tissues, and hence were included in our study to analyze their expression patterns across 6 tissue groups using a heatmap (Supplemental Fig. 16). While SLCO1B3 (OATP1B3), OATP2A1, OATP2B1, and SLC22A1 (OCT1) exhibited the highest expression in the liver, many SLCs, including OATP1A2, OATP2A1, OATP2B1, OATP1C1, OATP3A1, SLC22A5 (OCTN2), SLC22A6 (OAT1), SLC22A15 (fly-like putative transporter 1, FLIPT1), SLC22A17 (brain-type organic cation transporter, BOCT), and SLC22A23 had significant expression in various ocular subtissues. Additionally, SLC47A1 (MATE1) and OATP1B3 showed moderate expression in the RC.

Beyond the major drug-transporting SLCs, the SLC 1, 3, 6, 7, 10, 15, 28, 29, and 35 families were also reported to be involved in the transport of clinical drugs (Roberts, 2021). There are 69 such SLCs in the rabbit species, hence were also included to our study. The top 10 expressed SLCs within each tissue were identified using the averaged log_2_-transformed TPM values and visualized in the bar plots (Supplemental Fig. 17). The results highlighted that SLC3A2 was highly expressed across all 6 tissues, while SLC6A6, SLC7A5, and SLC22A17 were highly expressed across all the ocular subtissues, with SLC6A6 and SLC22A17 also showing high expression in the duodenum. Additionally, SLC6A9, SLC10A3, and SLC22A6 were highly expressed in most of the ocular subtissues. Several members of the SLC35 family, the nucleotide sugar transporters, showed prominent expression in the cornea, liver, and duodenum. Moreover, SLC1A5, a neutral amino acid transporter, was highly expressed in the cornea; SLC7A3, SLC7A8, and SLC22A5 were highly expressed in the ICB; SLC7A6 and SLC7A8 were highly expressed in the VH; and SLC1A3 and SLC29A1 were highly expressed in the RC. In contrast, liver showed high expression of OATP1B3, OATP2B1, SLC7A2, and SLC22A1, whereas duodenum showed high expression of SLC1A1, SLC15A1, and SLC28A2.

Furthermore, 15 SLC transporters with key roles in human drug transport and high expression in ocular subtissues (namely, ocular SLC transporters) were selected for quantitative comparative analysis using box-and-whisker plots (Supplemental Fig. 18). These included OATP1A2, OATP1B3, OATP1C1, OATP2A1, OATP2B1, OATP3A1, OCTN2, OAT1, FLIPT1, BOCT1, BOCT2, MATE1, SLC3A2, SLC6A6, and SLC7A5. Among these transporters, OAT1, OATP1C1, OATP3A1, FLIPT1, BOCT1, SLC3A2, SLC6A6, SLC7A5 and MATE1 exhibited higher or similar expression levels in all the ocular subtissues than in the liver or duodenum; OCTN2 showed similar expression level across all the tissues, except for the VH had lower expression level. OATP1A2 had comparably high expression levels in ICB and RC relative to the liver, and moderate expression levels in the cornea and VH, with minimal expression in the duodenum. OATP2A1, OATP2B1, and BOCT2 exhibited considerable expression levels in all the ocular subtissues with liver or duodenum showing the highest expression level. OATP1B3 showed the highest expression level in the liver, followed by the RC, and minimal expression in the other tissues.

### 3.9 Sex-dependent expression of DMEs and DTs

In our model that included a sex effect, we found that a total of 20/387 DME (Supplemental Fig. 19) and 10/128 DT (Supplemental Fig. 20) genes exhibited sex-dependent in the differences in expression between the ocular tissues and the liver (sex-by-tissue interaction *P*_*FDR*_ < .01; Supplemental Table 7). The most striking example of this difference is CYP2U1, which showed very little expression across all ocular tissues in both males and females, and low expression in the liver of males, but relatively high expression in the liver of females (Supplemental Fig. 19), suggesting that the patterns of differential expression we see between ocular and metabolic tissues is predominantly driven by this sex-dependent difference in the liver. Additionally, CYP2J1 and CYP39A1 showed striking sex differences in the liver (Supplemental Fig. 19), but for these genes the overall average expression was higher in the liver than the ocular tissues, regardless of the sex difference. There were several genes in which there were striking differences in the VH for males, such as ALDH16A1, ABCC10, SLC35G2 (Supplemental Figs. 19 and 20), however, there was also considerable variability among the male replicates for VH, potentially due to the challenges with RNA extraction from this tissue. Overall we observed that although there are some genes for which there is sex-dependence in the differential expression between ocular and metabolic tissues, the main difference between the tissues is larger overall and thus sex differences are unlikely to be the major driver of differences we report here.

### 3.10 Comparison to human ocular expression of DMEs and DTs

The Human Eye Transcriptome Atlas (Wolf et al, 2022) database was searched for the ocular DME and DT genes identified in our study (68 DMEs and 30 DTs), including 15 CYPs, 20 hydrolases, 14 non-CYPs oxidases, 10 reductases, 9 transferases, 15 ABC transporters, and 15 SLC transporters. All ocular genes were reportedly expressed in various human ocular tissues, except for CYP51A1, CES1, and NTAN1, which only had appreciable expression in several posterior ocular tissues or cells, eg, retinal microglia and hyalocytes. Although CES1 had little expression in human ocular tissues, its functional analog CES2 had moderate to high expression, a notable difference between humans and rabbits.

It is also important to note that the human ocular transcriptomics dataset from The Human Eye Transcriptome Atlas includes a total of 201 samples, of which, 133 samples were treated with formalin fixation and paraffin embedding (FFPE), and the rest of 68 samples were unfixed. The FFPE treatment has been reported to reduce mRNA levels by 85%–99% compared to matched frozen tissues (Abrahamsen et al, 2003). Because the cornea (n = 10), retina center (n = 3), retina periphery (n = 3), and choroid/ retinal pigment epithelium (RPE; n = 4) samples in the human dataset were all FFPE-treated, it presented a significant challenge to reliably parse the cross-tissue expression patterns and compare them to those of rabbits. Furthermore, no metabolic organ, such as liver or duodenum, was included for cross-tissue expression comparison in the human dataset. Given these limitations, it was impossible to carry out a systematic cross-species comparison between rabbits and humans. Nonetheless, 2 ocular DMEs were selected to demonstrate both consistent and inconsistent cross-tissue expression patterns between rabbits and humans. For example, CYP1B1 showed the highest expression in the rabbit ICB and RC, followed by VH, and little expression in the cornea (Fig. 4). In the human eye (healthy), CYP1B1 showed a similar pattern with highest expression in the optic nerve and retina center, moderate expression in the choroid/RPE, and no expression in the cornea. In contrast, CYP2S1 showed similar expression in the rabbit cornea, ICB, RC and VH (Fig. 4), whereas its expression in the human eye (healthy) was highly variable across different ocular tissues with highest expression in the conjunctiva, moderate expression in the cornea and little expression in the optic nerve, retina center or choroid/RPE.

## 4 Discussion

Our study provides a comprehensive view of DMEs and DTs in the rabbit eye compared to major metabolic organs such as the liver and duodenum. The gene expression profiles obtained confirms the liver’s dominant role in drug metabolism and disposition, showing the highest overall expression of many well known DMEs and DTs. Furthermore, our study confirms that the eye possesses considerable drug-metabolizing and drug-transporting capacities, challenging the traditional view of the eye as a minor metabolic organ and suggesting that ocular drug disposition should receive more attention than previously recognized. Ocular tissues exhibit distinct metabolic capabilities from the liver and duodenum, which may uniquely influence the ocular disposition of locally or systemically administered drugs.

The cytochrome P450 superfamily plays a crucial role in the metabolism of a wide range of xenobiotics, accounting for the metabolism of over 50% of clinically used drugs (Cerny, 2016). The low expression levels of CYP1A, CYP2C, and CYP3A in rabbit ocular tissues (Fig. 2) mirror the similarly low levels reported in human ocular tissues (Zhang et al, 2008; Wolf et al, 2022). These findings suggest potential interspecies conservation in the ocular P450 expression profiles between rabbits and humans. While the eye showed relatively low expression of hepatic CYPs, other CYPs such as CYP1B1, CYP2J1, CYP20A1, and CYP27C1 displayed high expression levels in ocular tissues (Fig. 4). These enzymes participate in the metabolism of endogenous compounds, such as sterols, vitamin A, and fatty acids (Esteves et al, 2021; Pikuleva, 2023). Notably, CYP1B1 had very high expression particularly in the ICB and RC, aligned with its known role in steroid metabolism and its association with glaucoma (Vasiliou and Gonzalez, 2008; Alsubait et al, 2020). The comparative analysis of CYPs reveal clear tissue-specific expression patterns (Fig. 2), suggesting the eye may possess unique metabolic capabilities for handling certain drugs, particularly those metabolized by non-hepatic CYP enzymes.

In addition to CYP enzymes, hydrolases are also critical in phase I drug metabolism, mediating the hydrolysis of a wide range of drugs and endogenous substrates. Approximately 20% of marketed drugs are susceptible to hydrolysis (Fukami et al, 2022), and hydrolases are responsible for activating nearly 50% of prodrugs (Di, 2019). Hydrolases are particularly important to the eye due to their role in the bioactivation of ocular drugs (Cerny, 2016; Argikar et al, 2017a). Hydrolases, such as CESs, AADAC, PONs, are critical for the hydrolysis of ester- and amide-containing drugs, and their high expression in the liver confirms the liver’s dominant role in biotransforming these substrates. Our study also confirmed the presence of CESs, PONs, AADAC, and GUSB in the ocular tissues, especially the high expression of CES1 in the cornea and ICB (Supplemental Fig. 1), consistent with previous reports (Kaluzhny et al, 2018; Hammid and Honkakoski, 2024). EPHX1 showed high expression across all ocular subtissues (Supplemental Fig. 1), suggesting protective functions against oxidative stress, which is particularly important in the eye as it is frequently exposed to environmental insults like UV light. FAAH, known for hydrolyzing fatty acid amides, has been implicated for regulating endocannabinoid signaling within the eye and modulating intraocular pressure, making it a target for glaucoma therapies (Spadoni et al, 2018; Miller et al, 2020). A striking finding from the hydrolase analysis is the high abundance of peptidases in the ocular tissues (Supplemental Figs. 1 and 2). This finding is important because that protein and peptide biologics are gaining popularity in treating ocular diseases such as diabetic retinopathy, glaucoma, and age-related macular degeneration (Mandal et al, 2018; Boddu et al, 2023). The high expression of peptidases in the eye has important implications for the design of peptide-based drugs or prodrugs, as these enzymes could influence the stability and bioavailability of such molecules.

Our study revealed that phase I reductases and non-CYP oxidases showed considerable expression in ocular tissues. For instance, ALDH1A1 and ADH5 were ubiquitously expressed in the ocular subtissues, liver, and duodenum (Supplemental Fig. 3), suggesting their key role in detoxification and local metabolic processes in these tissues. Additionally, the expression of antioxidant enzymes like GPX1, GPX2, and GPX3 across ocular subtissues (Supplemental Fig. 4) highlights the eye’s capacity to combat oxidative stress, critical for protecting metabolically active tissues like retina and cornea. The high expression of reductases like AKR1B1 and DHRS1 in various ocular subtissues highlights their relevance in detoxification and oxidative stress protection within ocular tissues. Furthermore, POR, a key reductase for cytochrome P450 enzymes, was abundantly expressed in the cornea, ICB, and RC (Supplemental Figs. 7 and 9), highlighting its importance in supporting the activity of CYP-mediated metabolism in ocular tissues.

Transferases are important contributors to the clearance of xenobiotics. UGTs, which are primary contributors to hepatic drug conjugation, showed minimal expression in ocular tissues. UGT1A6 and UGT8 was observed to have expression in the cornea and RC, respectively, but at much lower levels than in the liver (Supplemental Fig. 7). While literature on UGT activity in ocular tissues remains limited, the generally low expression of UGTs in the eye observed in this study could mean that ocular tissues have a low capacity for glucuronidation. In contrast, GSTs were the most prominent transferases across the ocular subtissues (Supplemental Fig. 8). GSTs play a crucial role in protection against oxidative stress and detoxifying a wide range of therapeutic drugs (Allocati et al, 2018). The high expression of GSTs across ocular subtissues suggests that the eye possesses detoxificaties capability toward xenobiotics and endogenous compounds, which defends the eye against oxidative damage. Additionally, the moderate expression of SULT1A1, SULT1C2, and SULT4A1 across the ocular subtissues underscores the potential for sulfate conjugation of drug molecules in the eye.

Drug transporters play an essential role in drug absorption, distribution, and excretion. In the context of ocular drug development and delivery, understanding the expression patterns of these transporters is crucial, as they can affect ocular drug bioavailability and toxicity, which are key parameters for evaluating the efficacy and safety during drug development (Vellonen et al, 2018). Moreover, evaluating the interactions of new drug molecules and certain transporters is also required during drug development (Huang et al, 2007). ABC transporters are primarily responsible for the active efflux of a wide range of substrates across cellular membranes, often acting as protective barriers against xenobiotics. Overall, the high expression of these efflux transporters in ocular tissues, especially in the blood vessel-enriched ICB and RC (Supplemental Figs. 13 and 15), highlights their importance in modulating drug distribution and penetration within the eye. The well known ABC DTs, eg, P-gp, MRPs, and BCRP, all exhibited high expression level in the ocular subtissues, especially in the ICB and RC, indicating their role in protecting the eye against foreign molecules from the systemic circulation and their involvement in the clearance of endogenous substrates and xenobiotics from ocular tissues. Several lesser-known ABC transporters were highly expressed in the eye. For example, ABCR plays a crucial role in the visual cycle by mediating the transport of retinal conjugate across photoreceptor disc membranes (Zhong et al, 2012); MTABC3 has been reported to regulate the expression and activity of some CYPs in mice (Chavan et al, 2015); and ABCG4 is known to play a role in the cellular efflux of desmosterol and amyloid-*β* peptide in the blood-brain barrier (BBB) (Dodacki et al, 2017), and possibly serves a similar role in the BRB of the eye. In summary, this active efflux capacity in the eye poses challenges for ocular drug delivery but also offers a protective mechanism for preventing drug accumulation in the eye and associated toxicity.

SLC transporters, in contrast to ABC transporters, generally facilitate the uptake of solutes, nutrients, and drugs into cells. Several OATPs, such as OATP1A2, OATP1C1, OATP2A1, OATP2B1, and OATP3A1, were highly expressed in the eye, liver or duodenum (Supplemental Figs. 16 and 18), suggesting similar uptake capacities of these ocular tissues to hepatic tissues. These OATPs were also reported to be expressed in the BBB (Konig, 2011). The high abundance of OAT1, OCTN2, and BOCTs in various ocular subtissues suggests that the eye may have significant drug uptake potential for organic anions, cations, and zwitterions. Interestingly, MATE1, the organic cation antiporter (Yonezawa and Inui, 2011), exhibited particularly high expression in the RC (Supplemental Fig. 18), suggesting its role in excluding organic cations from the eye. For ocular drug development, targeting transporters to either enhance drug uptake or inhibit efflux represents a promising strategy. Drugs designed to avoid efflux by ABC transporters or to selectively utilize SLC transporters for uptake may have improved bioavailability and efficacy in treating ocular diseases. Future studies should focus on understanding the interaction between ocular DTs and specific therapeutic agents, aiding the development of more effective ocular therapeutics.

The differential expression between liver and ocular tissues we observe for some of the CYPs, such as CYP2U1, CYP2J1, and CYP39A1, appears largely driven by sexually-dimorphic variation in expression in the liver. CYP2U1 and CYP2J1 were expressed higher in the liver of females, minimal or nearly no expression in the liver of males or any of the ocular tissues, whereas CYP39A1 had higher expression in the liver of males. In mice, CYP2U1 was primarily detected in male liver, ∼330% higher in male liver than in female; CYP2J1 were expressed higher in the female liver than in male (Renaud et al, 2011). Although there were some other DMEs and DTs that exhibited significant sex-by-tissue interactions in the contrast of ocular and liver tissue, it was only the case with these CYP genes that the main effect of the tissue comparison may be predominantly driven by sex-based differences in liver expression.

In conclusion, this study provides a comprehensive characterization of the expression profiles of DMEs and DTs in the rabbit ocular subtissues, liver, and duodenum, contributing to our understanding of drug metabolism and disposition in the eye. These findings are expected to aid future research aimed at optimizing ocular drug delivery systems, improving interspecies extrapolation between rabbits and humans, and expediting ophthalmic drug development.

## Conflict of interest

The authors declare no conflicts of interest.

## References

[bib1] Abrahamsen H.N., Steiniche T., Nexo E., Hamilton-Dutoit S.J., Sorensen B.S. (2003). Towards quantitative mRNA analysis in paraffin-embedded tissues using real-time reverse transcriptase-polymerase chain reaction: a methodological study on lymph nodes from melanoma patients. J Mol Diagn.

[bib2] Allocati N., Masulli M., Di Ilio C., Federici L. (2018). Glutathione transferases: substrates, inihibitors and pro-drugs in cancer and neurodegenerative diseases. Oncogenesis.

[bib3] Almazroo O.A., Miah M.K., Venkataramanan R. (2017). Drug metabolism in the liver. Clin Liver Dis.

[bib4] Alsubait A., Aldossary W., Rashid M., Algamdi A., Alrfaei B.M. (2020). CYP1B1 gene: Implications in glaucoma and cancer. J Cancer.

[bib5] Argikar U.A., Dumouchel J.L., Dunne C.E., Bushee A.J. (2017). Ocular non-P450 oxidative, reductive, hydrolytic, and conjugative drug metabolizing enzymes. Drug Metab Rev.

[bib6] Argikar U.A., Dumouchel J.L., Kramlinger V.M., Cirello A.L., Gunduz M., Dunne C.E., Sohal B. (2017). Do we need to study metabolism and distribution in the eye: why, when, and are we there yet?. J Pharm Sci.

[bib7] Boddu S.H.S., Acharya D., Hala V., Jani H., Pande S., Patel C., Shahwan M., Jwala R., Ranch K.M. (2023). An update on strategies to deliver protein and peptide drugs to the eye. ACS Omega.

[bib8] GBD 2019 Blindness and Vision Impairment Collaborators, Vision Loss Expert Group of the Global Burden of Disease Study (2021). Trends in prevalence of blindness and distance and near vision impairment over 30 years: an analysis for the Global Burden of Disease Study. Lancet Glob Health.

[bib9] Cerny M.A. (2016). Prevalence of non-cytochrome p450-mediated metabolism in food and drug administration-approved oral and intravenous drugs: 2006–2015. Drug Metab Dispos.

[bib10] Chavan H., Li F., Tessman R., Mickey K., Dorko K., Schmitt T., Kumer S., Gunewardena S., Gaikwad N., Krishnamurthy P. (2015). Functional coupling of ATP-binding cassette transporter Abcb6 to cytochrome P450 expression and activity in liver. J Biol Chem.

[bib11] Debose-Boyd R.A. (2007). A helping hand for cytochrome p450 enzymes. Cell Metab.

[bib12] Di L. (2019). The impact of carboxylesterases in drug metabolism and pharmacokinetics. Curr Drug Metab.

[bib13] Di Tommaso P., Chatzou M., Floden E.W., Barja P.P., Palumbo E., Notredame C. (2017). Nextflow enables reproducible computational workflows. Nat Biotechnol.

[bib14] Dobin A., Davis C.A., Schlesinger F., Drenkow J., Zaleski C., Jha S., Batut P., Chaisson M., Gingeras T.R. (2013). STAR: ultrafast universal RNA-seq aligner. Bioinformatics.

[bib15] Dodacki A., Wortman M., Saubamea B., Chasseigneaux S., Nicolic S., Prince N., Lochus M., Raveu A.L., Decleves X., Scherrmann J.M. (2017). Expression and function of Abcg4 in the mouse blood-brain barrier: role in restricting the brain entry of amyloid-beta peptide. Sci Rep.

[bib16] Esteves F., Rueff J., Kranendonk M. (2021). The central role of cytochrome p450 in xenobiotic metabolism-a brief review on a fascinating enzyme family. J Xenobiot.

[bib17] Ewels P.A., Peltzer A., Fillinger S., Patel H., Alneberg J., Wilm A., Garcia M.U., Di Tommaso P., Nahnsen S. (2020). The nf-core framework for community-curated bioinformatics pipelines. Nat Biotechnol.

[bib18] Feng J., Sun J., Wang M.Z., Zhang Z., Kim S.T., Zhu Y., Sun J., Xu J. (2010). Compilation of a comprehensive gene panel for systematic assessment of genes that govern an individual's drug responses. Pharmacogenomics.

[bib19] Fukami T., Yokoi T., Nakajima M. (2022). Non-P450 drug-metabolizing enzymes: contribution to drug disposition, toxicity, and development. Annu Rev Pharmacol Toxicol.

[bib20] Galili T. (2015). dendextend: an R package for visualizing, adjusting and comparing trees of hierarchical clustering. Bioinformatics.

[bib21] Gaudana R., Ananthula H.K., Parenky A., Mitra A.K. (2010). Ocular drug delivery. AAPS J.

[bib22] Hammid A., Honkakoski P. (2024). Ocular drug-metabolizing enzymes: focus on esterases. Drug Metab Rev.

[bib23] Huang S.M., Temple R., Throckmorton D.C., Lesko L.J. (2007). Drug interaction studies: study design, data analysis, and implications for dosing and labeling. Clin Pharmacol Ther.

[bib24] ICCVAM (2010).

[bib25] Iyanagi T. (2007). Molecular mechanism of phase I and phase II drug-metabolizing enzymes: implications for detoxification. Int Rev Cytol.

[bib26] Jancova P., Anzenbacher P., Anzenbacherova E. (2010). Phase II drug metabolizing enzymes. Biomed Pap Med Fac Univ Palacky Olomouc Czech Repub.

[bib27] Kaluzhny Y., Kinuthia M.W., Truong T., Lapointe A.M., Hayden P., Klausner M. (2018). New human organotypic corneal tissue model for ophthalmic drug delivery studies. Invest Ophthalmol Vis Sci.

[bib28] Karlsson M., Zhang C., Mear L., Zhong W., Digre A., Katona B., Sjostedt E., Butler L., Odeberg J., Dusart P. (2021). A single-cell type transcriptomics map of human tissues. Sci Adv.

[bib29] Konig J. (2011). Uptake transporters of the human OATP family: molecular characteristics, substrates, their role in drug-drug interactions, and functional consequences of polymorphisms. Handb Exp Pharmacol.

[bib61] Krassowski M., Arts M., Lagger C., Max (2020). krassowski/complex-upset: v1.3.5 (v1.3.5).

[bib31] Krueger F., James F., Ewels P., Afyounian E., Schuster-Boeckler B. (2021). *TrimGalore: v0.6.7* [software]. Zenodo.

[bib32] Lex A., Gehlenborg N., Strobelt H., Vuillemot R., Pfister H. (2014). UpSet: visualization of intersecting sets. IEEE Trans Vis Comput Graph.

[bib33] Li B., Dewey C.N. (2011). RSEM: accurate transcript quantification from RNA-Seq data with or without a reference genome. BMC Bioinformatics.

[bib34] Love M.I., Anders S., Kim V., Huber W. (2015). RNA-Seq workflow: gene-level exploratory analysis and differential expression. F1000Res.

[bib35] Love M.I., Huber W., Anders S. (2014). Moderated estimation of fold change and dispersion for RNA-seq data with DESeq2. Genome Biol.

[bib36] Mandal A., Pal D., Agrahari V., Trinh H.M., Joseph M., Mitra A.K. (2018). Ocular delivery of proteins and peptides: challenges and novel formulation approaches. Adv Drug Deliv Rev.

[bib37] Manikandan P., Nagini S. (2018). Cytochrome p450 structure, function and clinical significance: A Review. Curr Drug Targets.

[bib38] Miller S., Daily L., Dharla V., Gertsch J., Malamas M.S., Ojima I., Kaczocha M., Ogasawara D., Straiker A. (2020). Endocannabinoid metabolism and transport as targets to regulate intraocular pressure. Exp Eye Res.

[bib39] Nakano M., Lockhart C.M., Kelly E.J., Rettie A.E. (2014). Ocular cytochrome P450s and transporters: roles in disease and endobiotic and xenobiotic disposition. Drug Metab Rev.

[bib46] Nath S., Das S., Kar o., Afrin K., Dash A., Akter S. (2016). Topographical and biometrical anatomy of the digestive tract of White New Zealand Rabbit (Oryctolagus cuniculus). J Adv Vet Anim Res.

[bib40] Petzinger E., Geyer J. (2006). Drug transporters in pharmacokinetics. Naunyn Schmiedebergs Arch Pharmacol.

[bib41] Pikuleva I.A. (2023). Challenges and opportunities in P450 research on the eye. Drug Metab Dispos.

[bib42] R Core Team (2023).

[bib43] Renaud H.J., Cui J.Y., Khan M., Klaassen C.D. (2011). Tissue distribution and gender-divergent expression of 78 cytochrome P450 mRNAs in mice. Toxicol Sci.

[bib44] Roberts A.G. (2021). The structure and mechanism of drug transporters. Methods Mol Biol.

[bib45] Rosati D., Palmieri M., Brunelli G., Morrione A., Iannelli F., Frullanti E., Giordano A. (2024). Differential gene expression analysis pipelines and bioinformatic tools for the identification of specific biomarkers: A review. Comput Struct Biotechnol J.

[bib47] Spadoni G., Bedini A., Furiassi L., Mari M., Mor M., Scalvini L., Lodola A., Ghidini A., Lucini V., Dugnani S. (2018). Identification of bivalent ligands with melatonin receptor agonist and fatty acid amide hydrolase (FAAH) inhibitory activity that exhibit ocular hypotensive effect in the rabbit. J Med Chem.

[bib48] Vasiliou V., Gonzalez F.J. (2008). Role of CYP1B1 in glaucoma. Annu Rev Pharmacol Toxicol.

[bib49] Vellonen K.S., Hellinen L., Mannermaa E., Ruponen M., Urtti A., Kidron H. (2018). Expression, activity and pharmacokinetic impact of ocular transporters. Adv Drug Deliv Rev.

[bib50] Wolf J., Boneva S., Schlecht A., Lapp T., Auw-Haedrich C., Lagreze W., Agostini H., Reinhard T., Schlunck G., Lange C. (2022). The human eye transcriptome atlas: a searchable comparative transcriptome database for healthy and diseased human eye tissue. Genomics.

[bib51] Yonezawa A., Inui K. (2011). Importance of the multidrug and toxin extrusion MATE/SLC47A family to pharmacokinetics, pharmacodynamics/toxicodynamics and pharmacogenomics. Br J Pharmacol.

[bib52] Zanger U.M., Schwab M. (2013). Cytochrome P450 enzymes in drug metabolism: regulation of gene expression, enzyme activities, and impact of genetic variation. Pharmacol Ther.

[bib53] Zernii E.Y., Baksheeva V.E., Iomdina E.N., Averina O.A., Permyakov S.E., Philippov P.P., Zamyatnin A.A., Senin I.I. (2016). Rabbit models of ocular diseases: new relevance for classical approaches. CNS Neurol Disord Drug Targets.

[bib54] Zhang T., Xiang C.D., Gale D., Carreiro S., Wu E.Y., Zhang E.Y. (2008). Drug transporter and cytochrome P450 mRNA expression in human ocular barriers: implications for ocular drug disposition. Drug Metab Dispos.

[bib55] Zhang Z., Tang W. (2018). Drug metabolism in drug discovery and development. Acta Pharm Sin B.

[bib56] Zhao M., Ma J., Li M., Zhang Y., Jiang B., Zhao X., Huai C., Shen L., Zhang N., He L., Qin S. (2021). Cytochrome P450 Enzymes and Drug Metabolism in Humans. Int J Mol Sci.

[bib57] Zhao Y., Li M.C., Konate M.M., Chen L., Das B., Karlovich C., Williams P.M., Evrard Y.A., Doroshow J.H., McShane L.M. (2021). TPM, FPKM, or normalized counts? A comparative study of quantification measures for the analysis of RNA-seq data from the NCI patient-derived models repository. J Transl Med.

[bib58] Zhong M., Kawaguchi R., Kassai M., Sun H. (2012). Retina, retinol, retinal and the natural history of vitamin A as a light sensor. Nutrients.

[bib59] Zhu A., Ibrahim J.G., Love M.I. (2019). Heavy-tailed prior distributions for sequence count data: removing the noise and preserving large differences. Bioinformatics.

