# Supplementary material for: Comparative transcriptomic profiling of drug-metabolizing enzymes and drug transporters in rabbit ocular subtissues, liver, and duodenum
Source: Drug Metab Dispos. 2025 May 20;53(7):100099. doi: 10.1016/j.dmd.2025.100099 (PMC12405909; doi:10.1016/j.dmd.2025.100099)
Supplement: Supplementary Figures 1-20 [file mmc8.pdf]

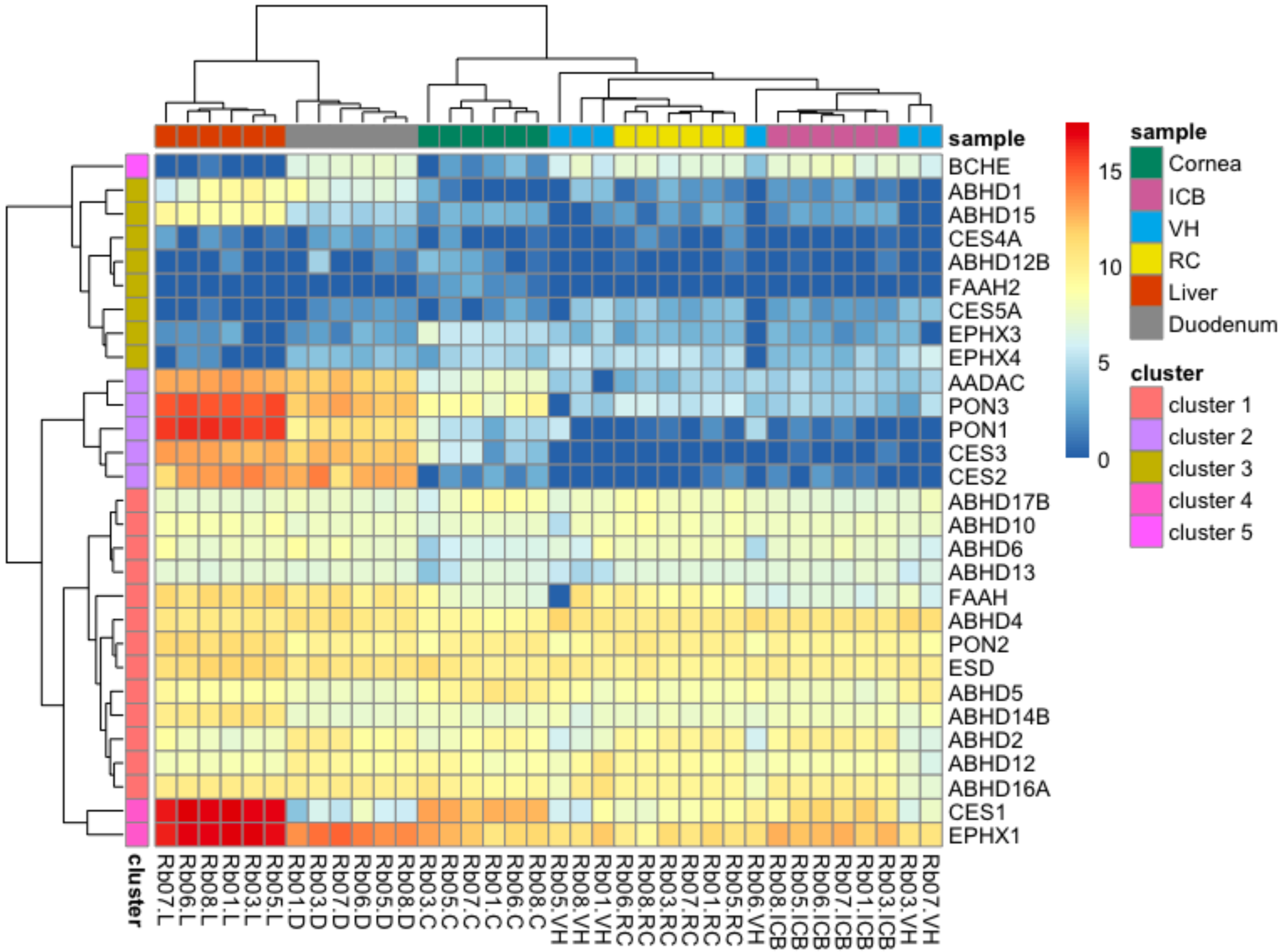

Supplemental Figure 1. The mRNA gene expression heatmap of major hydrolases (n=29) in rabbit ocular sub-tissues, liver, and duodenum. The log2-transformed DESeq2 normalized counts were used to generate the heatmap and results were clustered as described in the Materials and Methods.

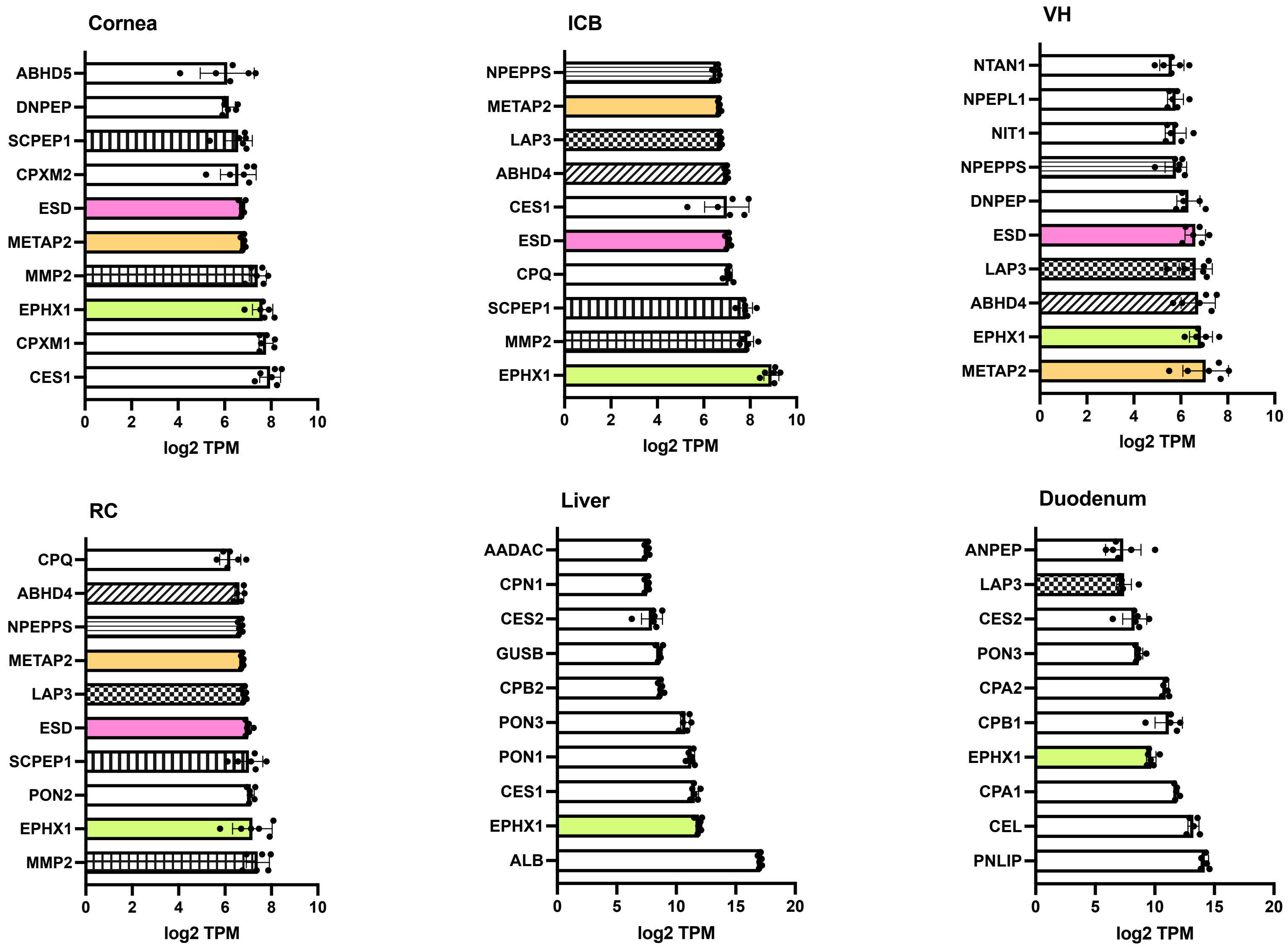

Supplemental Figure 2. Top 10 most abundantly expressed hydrolases genes in the rabbit cornea, ICB, VH, RC, liver, and duodenum. The log2-transformed transcripts per million (TPM) values were used to rank the expression within each tissue type. Genes identified in the top-10 lists of multiple tissue types were color/pattern-coded to facilitate visual identification.

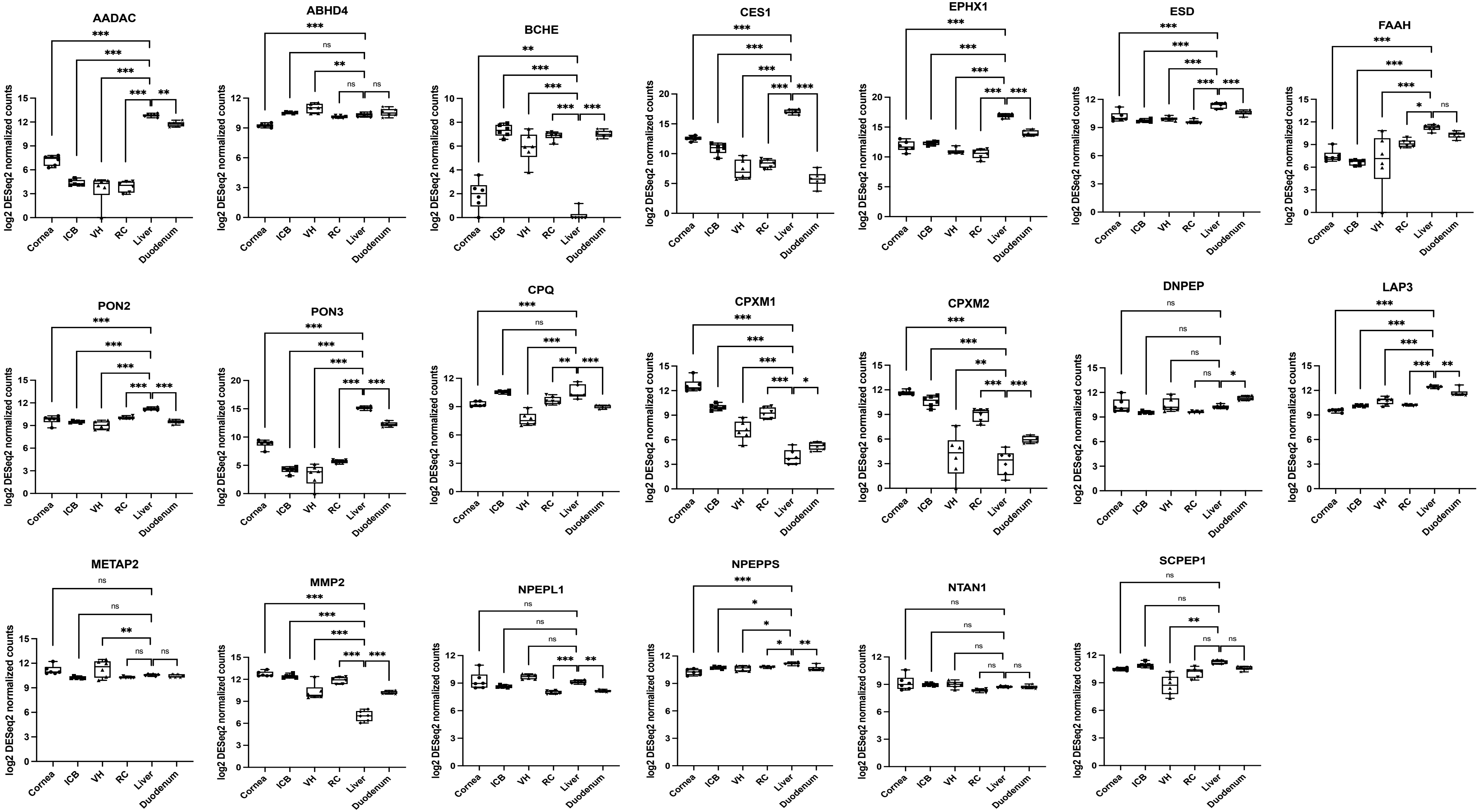

Supplemental Figure 3. The relative expression levels of ocular hydrolases genes in the rabbit cornea, ICB, VH, RC, liver, and duodenum. The $\log_2$-transformed DESeq2 normalized counts were used to generate box and whiskers plots, and the B-H adjusted P-values from DESeq2 were employed to compare the gene expression levels in the ocular sub-tissues and duodenum to the liver, as described in the Materials and Methods.

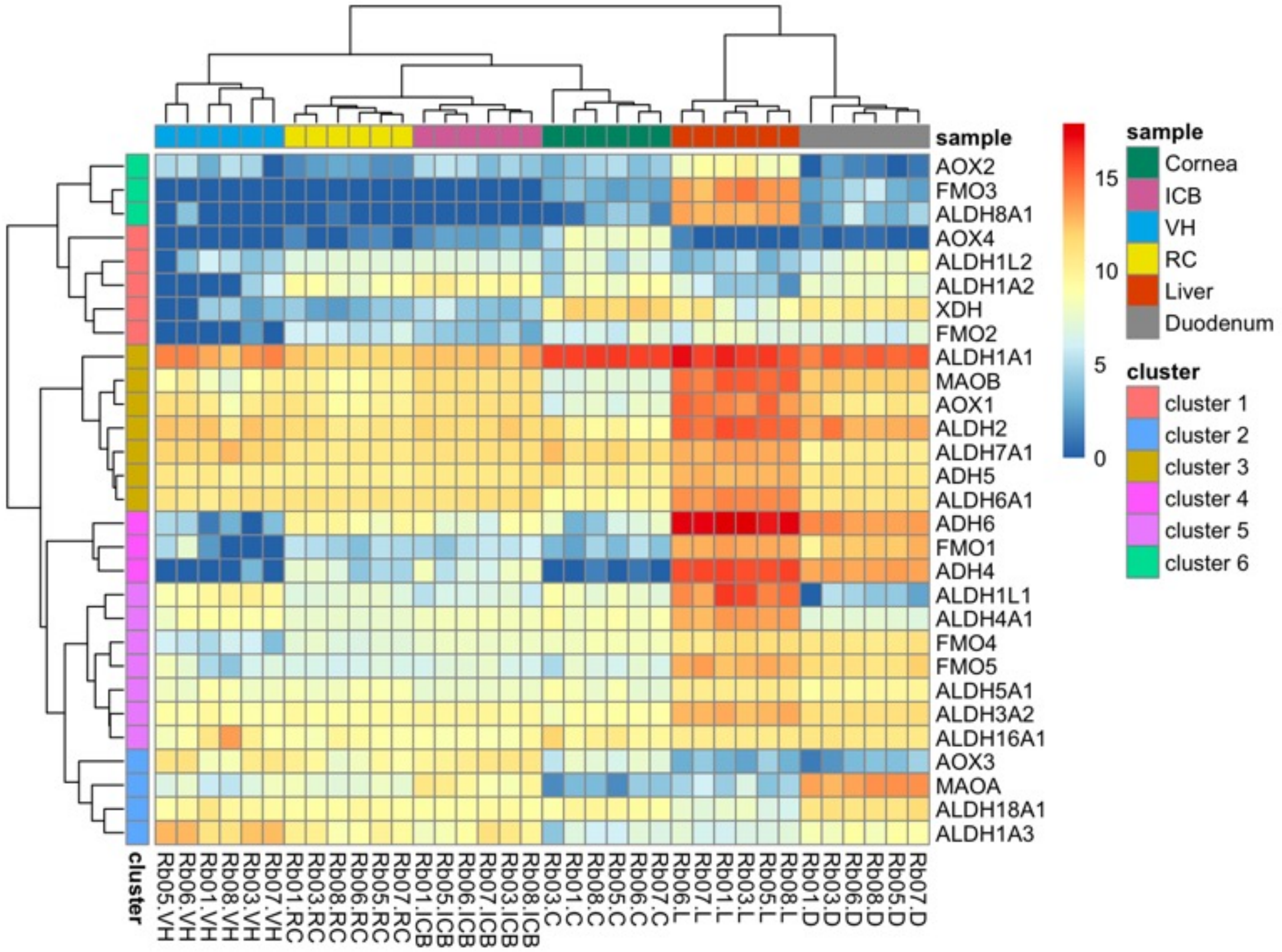

Supplemental Figure 4. The mRNA gene expression heatmap of major non-CYP oxidases (n=29) in rabbit ocular sub-tissues, liver, and duodenum. The log2-transformed DESeq2 normalized counts were used to generate the heatmap and results were clustered as described in the Materials and Methods.

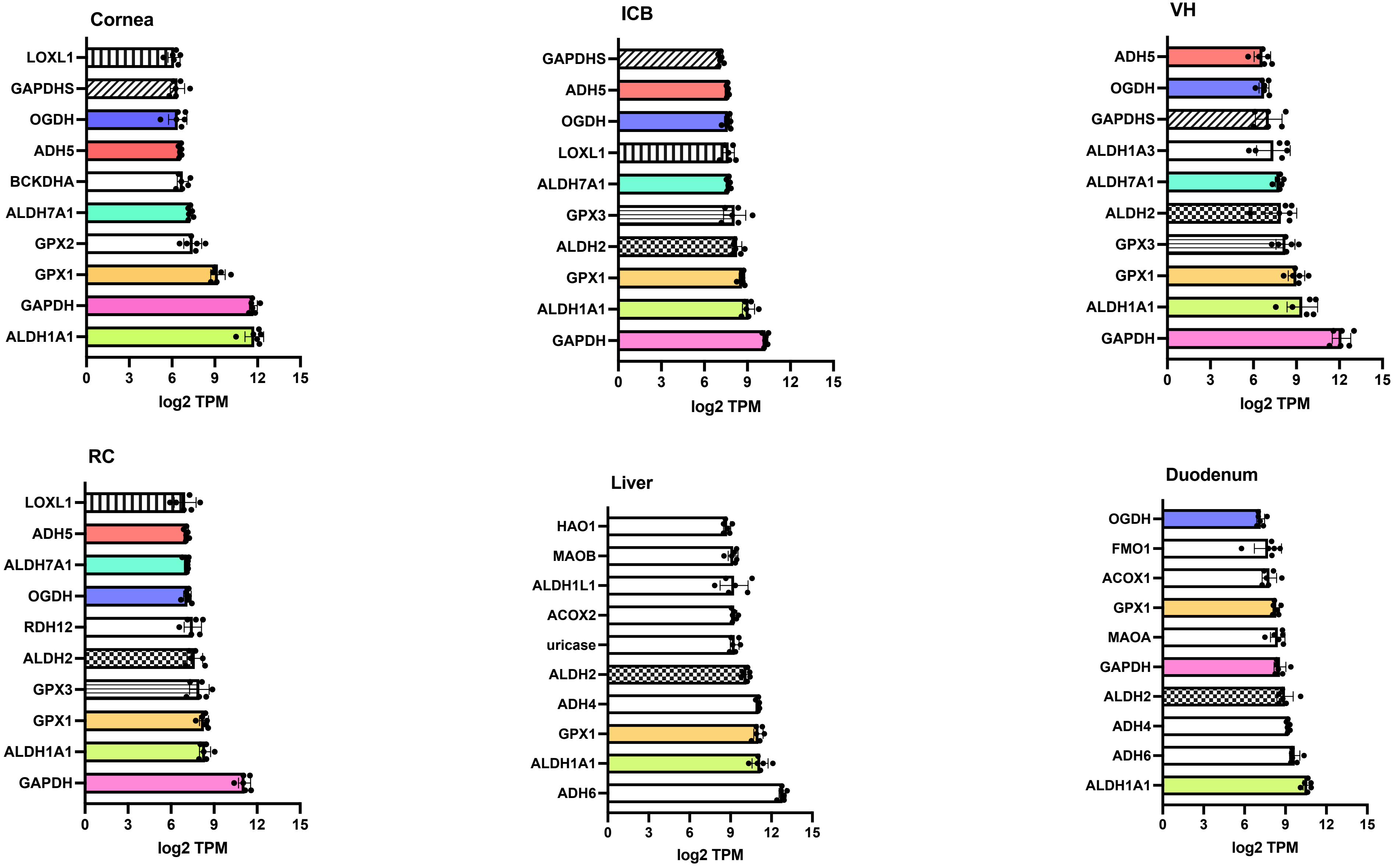

Supplemental Figure 5. Top 10 most abundantly expressed non-CYP oxidases genes in the rabbit cornea, ICB, VH, RC, liver, and duodenum. The log2-transformed transcripts per million (TPM) values were used to rank the expression within each tissue type. Genes identified in the top-10 lists of multiple tissue types were color/pattern-coded to facilitate visual identification.

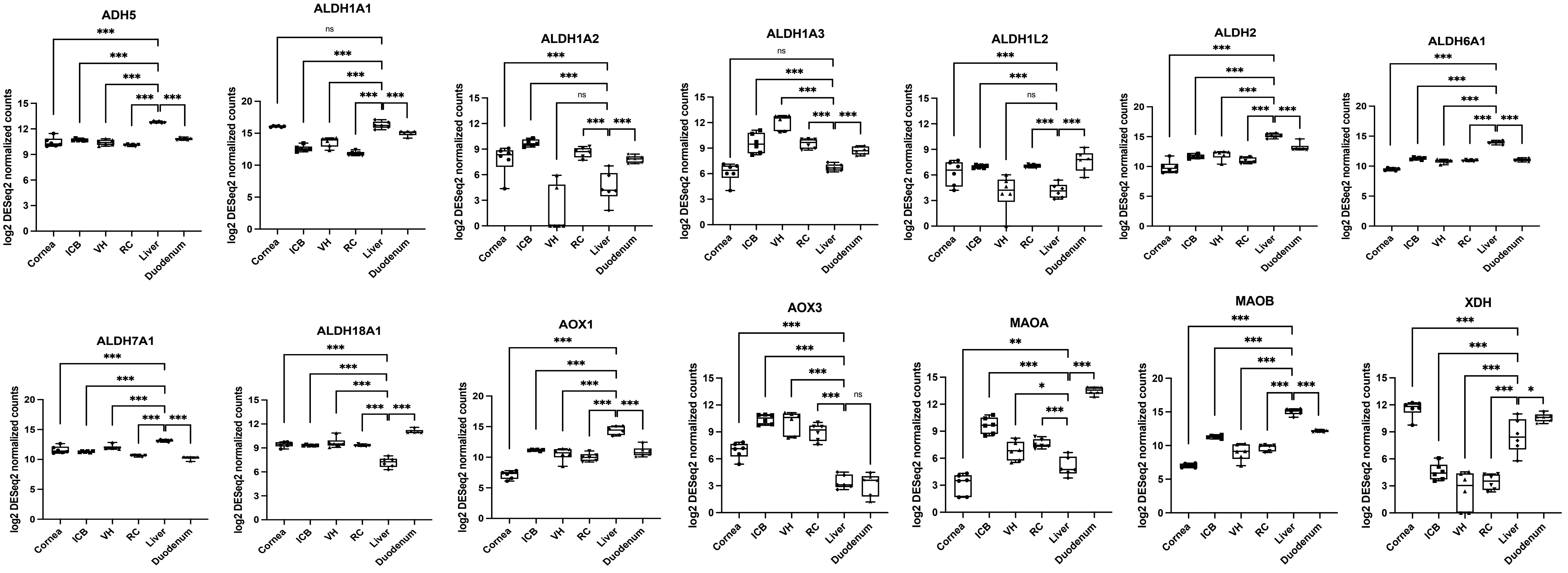

Supplemental Figure 6. The relative expression levels of ocular non-CYP oxidases in the rabbit cornea, ICB, VH, RC, liver, and duodenum. The log_2-transformed DESeq2 normalized counts were used to generate box and whiskers plots, and the B-H adjusted P-values from DESeq2 were employed to compare the gene expression levels in the ocular sub-tissues and duodenum to the liver, as described in the Materials and Methods.

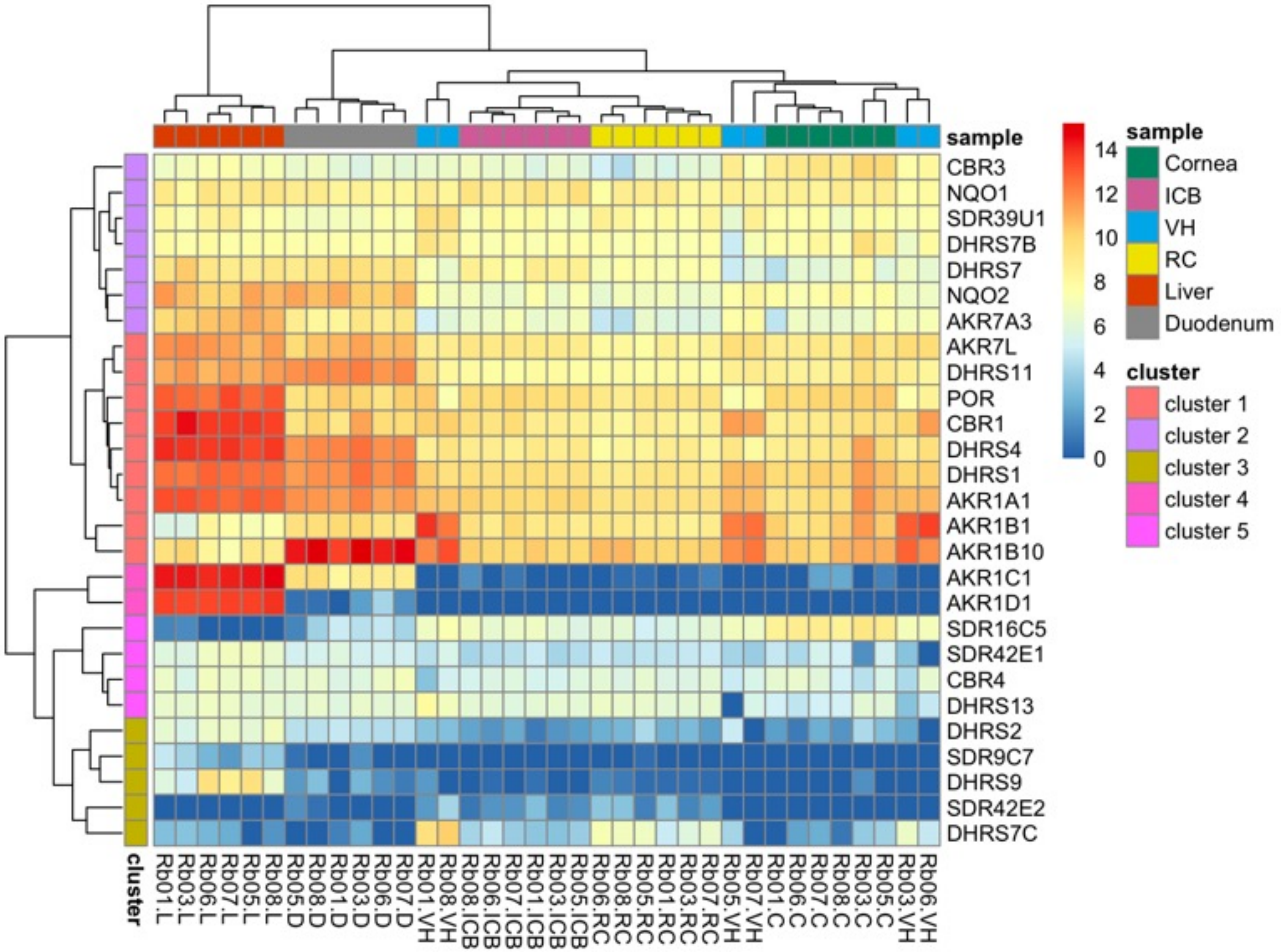

Supplemental Figure 7. The mRNA gene expression heatmap of major reductases (n=28) in rabbit ocular sub-tissues, liver, and duodenum. The log2-transformed DESeq2 normalized counts were used to generate the heatmap and results were clustered as described in the Materials and Methods.

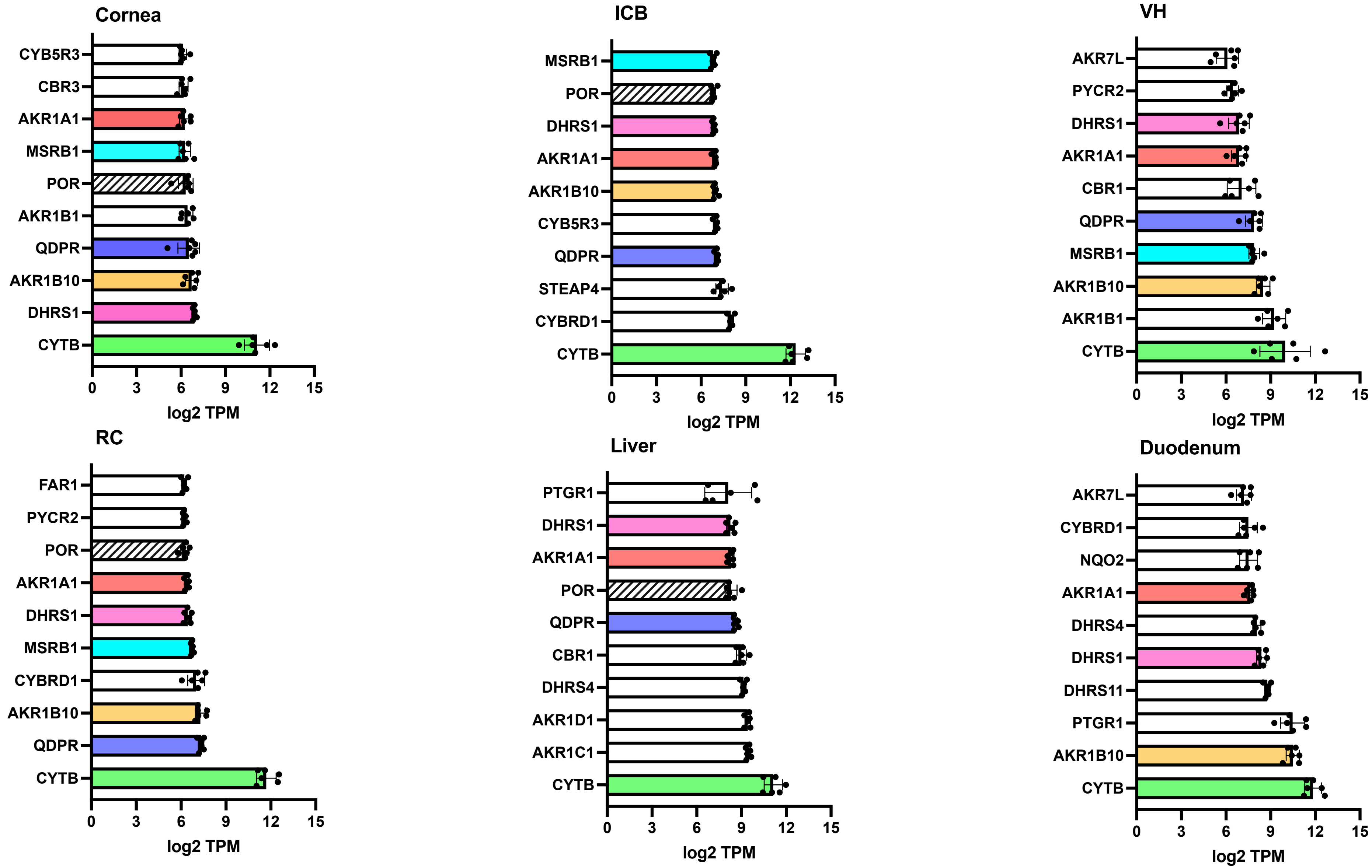

Supplemental Figure 8. Top 10 most abundantly expressed reductases genes in the rabbit cornea, ICB, VH, RC, liver, and duodenum. The log2-transformed transcripts per million (TPM) values were used to rank the expression within each tissue type. Genes identified in the top-10 lists of multiple tissue types were color/pattern-coded to facilitate visual identification.

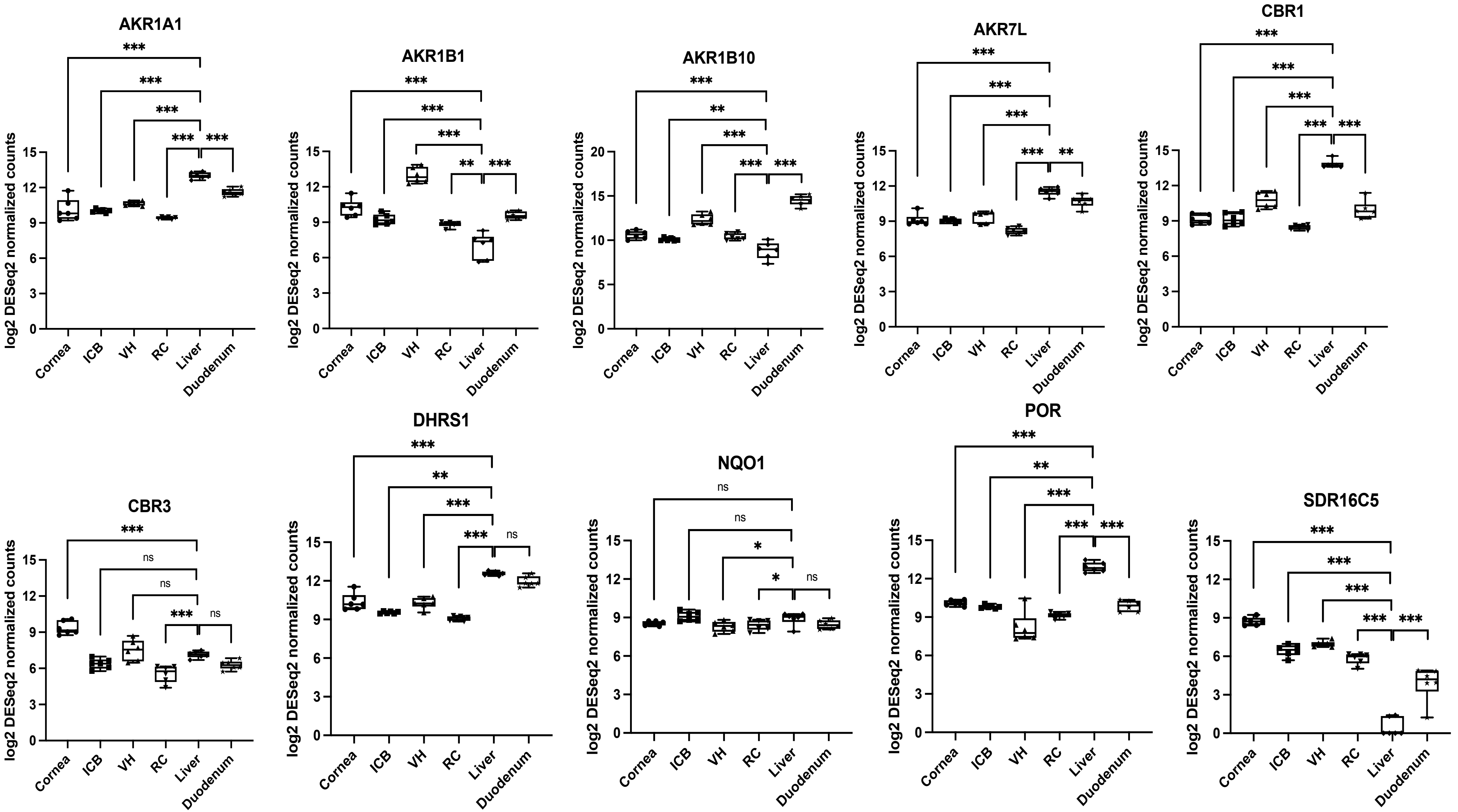

Supplemental Figure 9. The relative expression levels of ocular reductases in the rabbit cornea, ICB, VH, RC, liver, and duodenum. The log_2-transformed DESeq2 normalized counts were used to generate box and whiskers plots, and the B-H adjusted P-values from DESeq2 were employed to compare the gene expression levels in the ocular sub-tissues and duodenum to the liver, as described in the Materials and Methods.

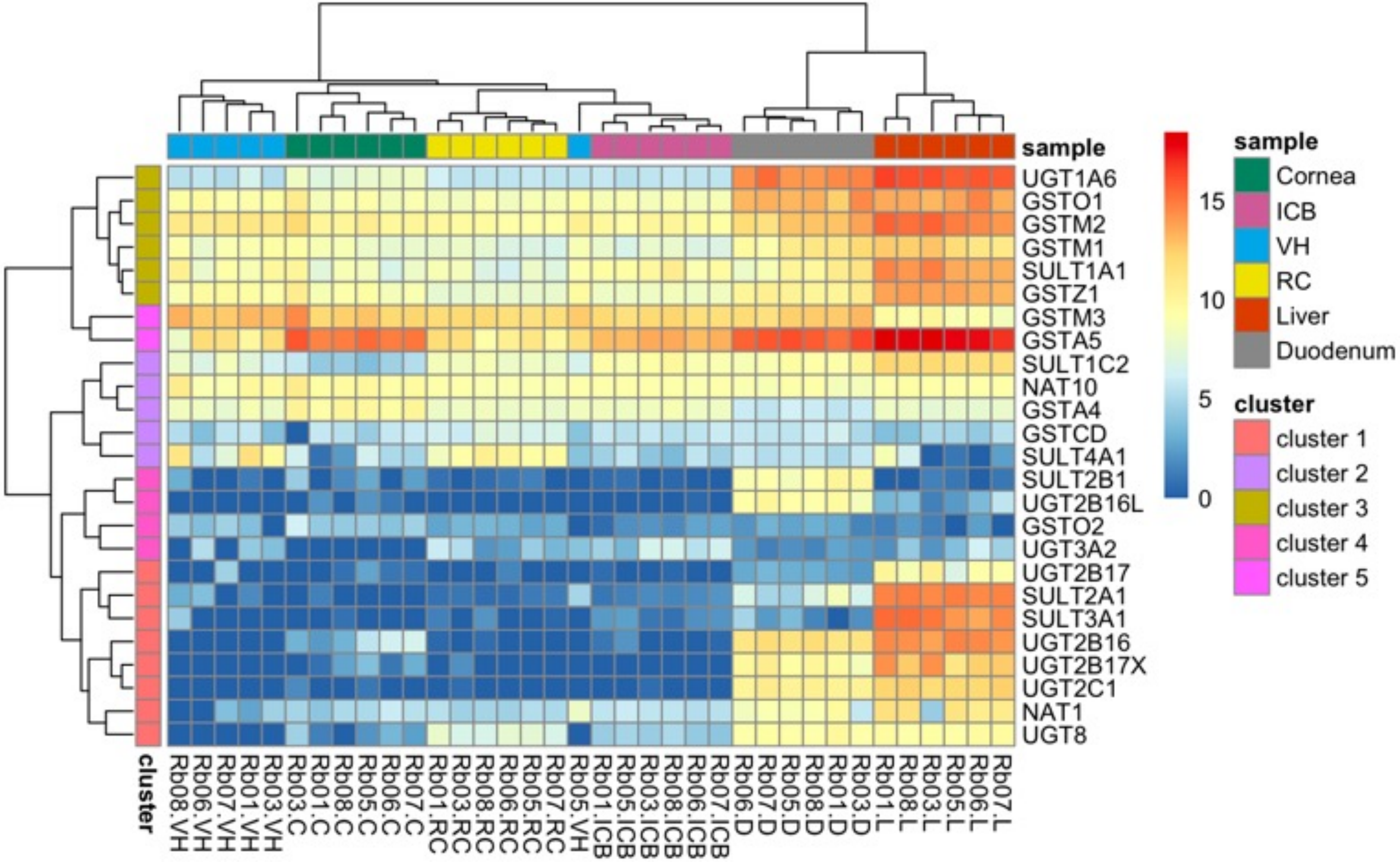

Supplemental Figure 10. The mRNA gene expression heatmap of major transferases (n=25) in rabbit ocular sub-tissues, liver, and duodenum. The log2-transformed DESeq2 normalized counts were used to generate the heatmap and results were clustered as described in the Materials and Methods.

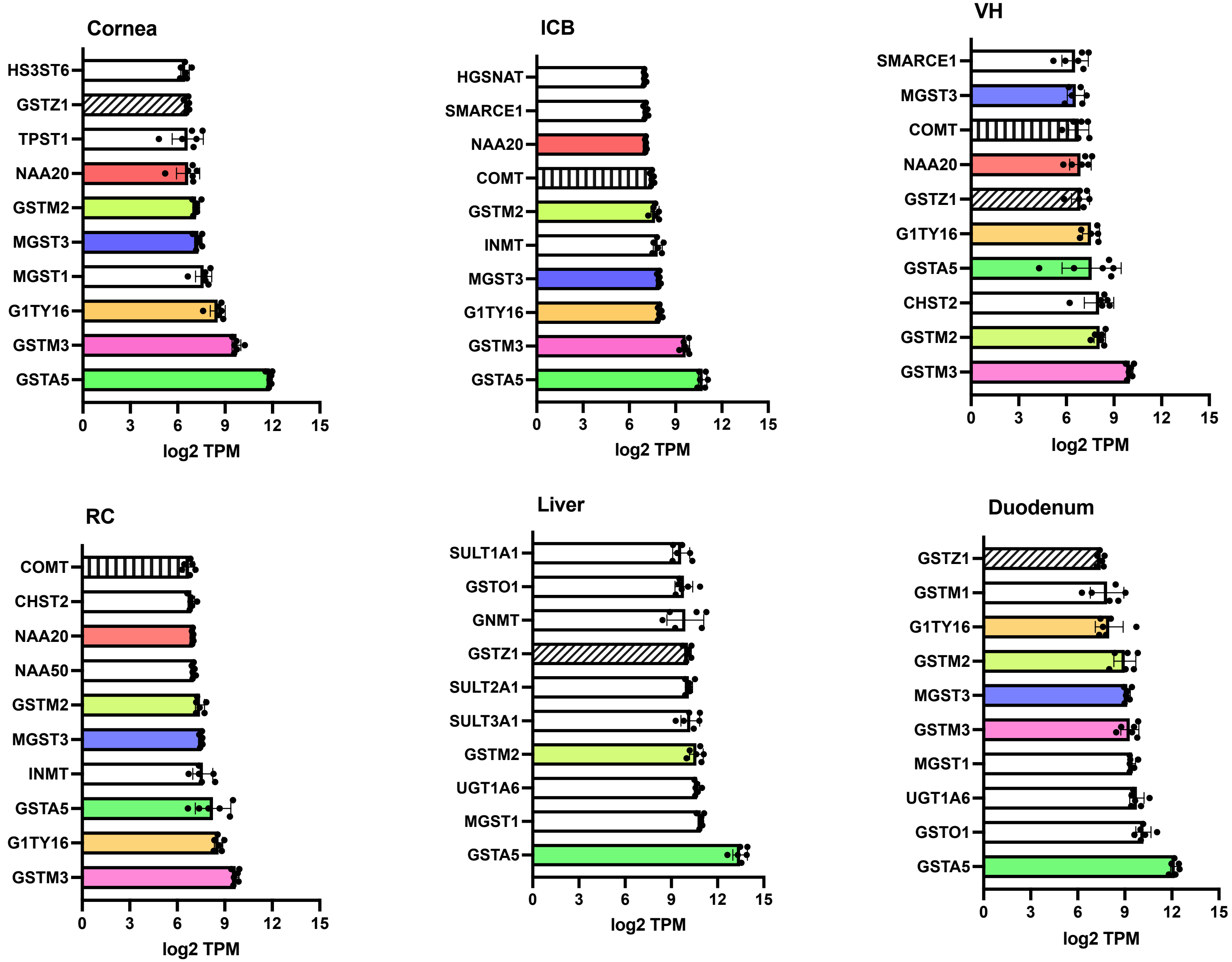

Supplemental Figure 11. Top 10 most abundantly expressed transferases genes in the rabbit cornea, ICB, VH, RC, liver, and duodenum. The log2-transformed transcripts per million (TPM) values were used to rank the expression within each tissue type. Genes identified in the top-10 lists of multiple tissue types were color/pattern-coded to facilitate visual identification.

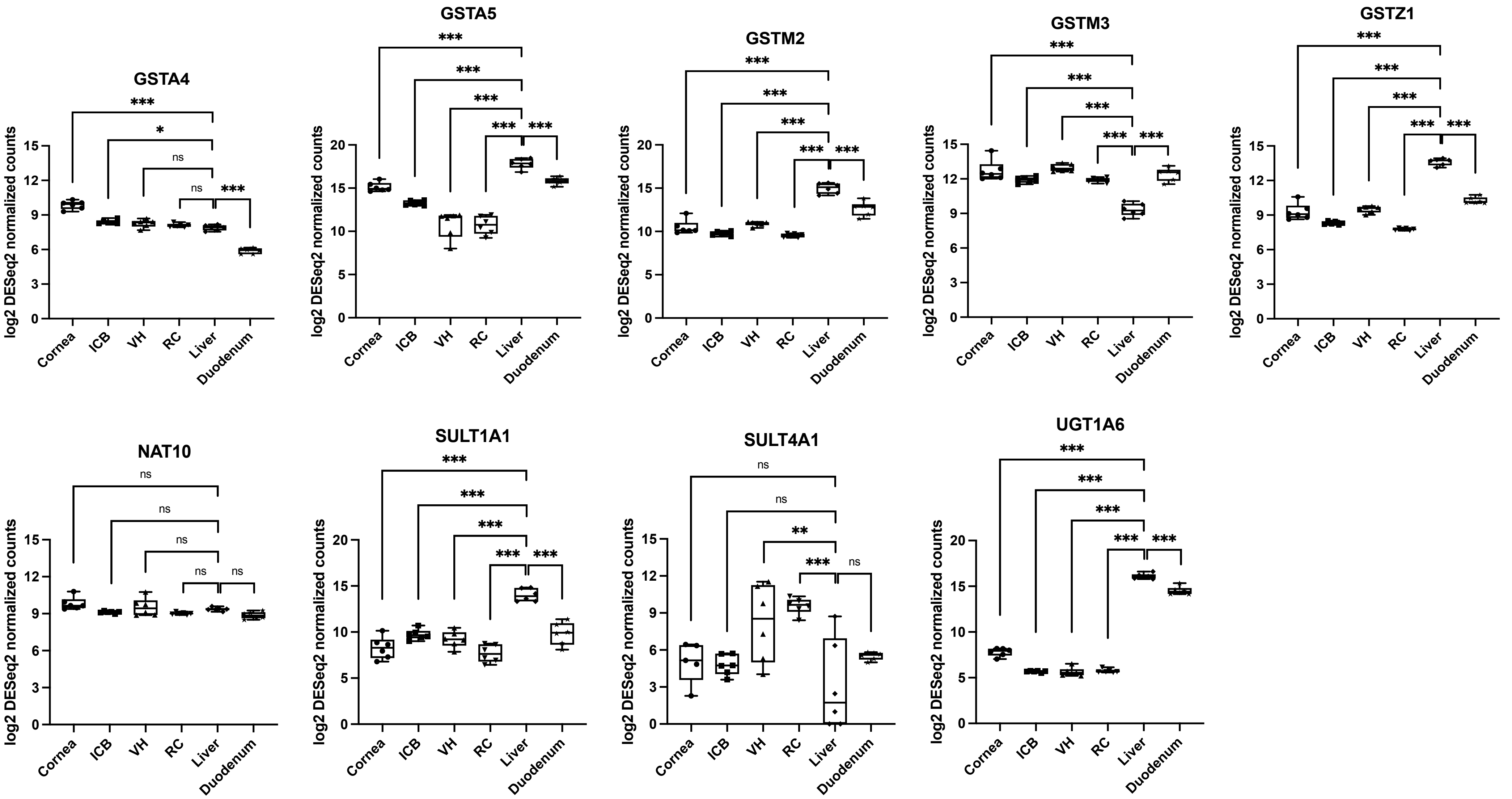

Supplemental Figure 12. The relative expression levels of ocular transferases in the rabbit cornea, ICB, VH, RC, liver, and duodenum. The $\log_2$-transformed DESeq2 normalized counts were used to generate box and whiskers plots, and the B-H adjusted P-values from DESeq2 were employed to compare the gene expression levels in the ocular sub-tissues and duodenum to the liver, as described in the Materials and Methods.

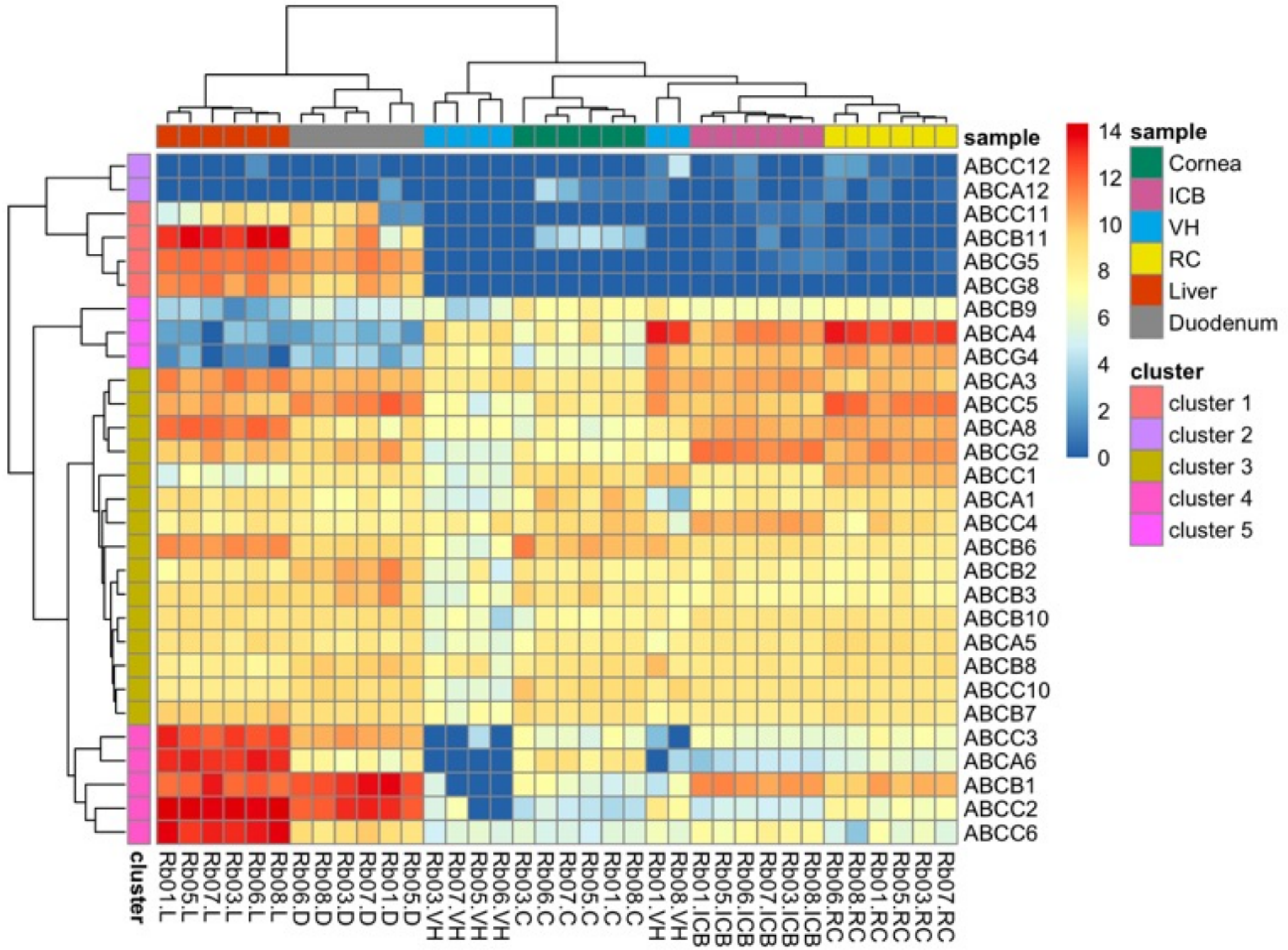

Supplemental Figure 13. The mRNA gene expression heatmap of major ABC transporters (n=29) in rabbit ocular sub-tissues, liver, and duodenum. The log2-transformed DESeq2 normalized counts were used to generate the heatmap and results were clustered as described in the Materials and Methods.

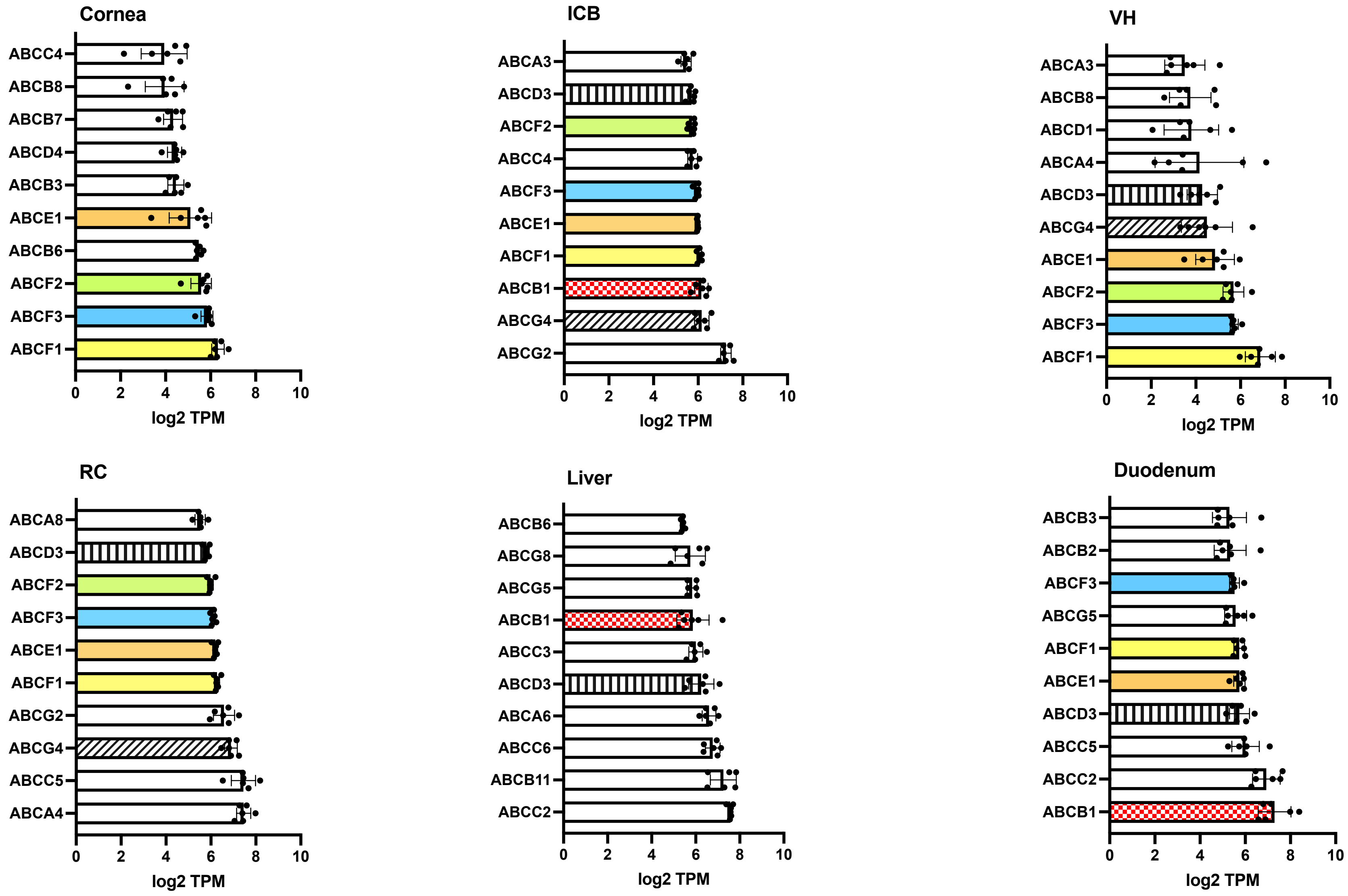

Supplemental Figure 14. Top 10 most abundantly expressed ABC transporters genes in the rabbit cornea, ICB, VH, RC, liver, and duodenum. The log2-transformed transcripts per million (TPM) values were used to rank the expression within each tissue type. Genes identified in the top-10 lists of multiple tissue types were color/pattern-coded to facilitate visual identification.

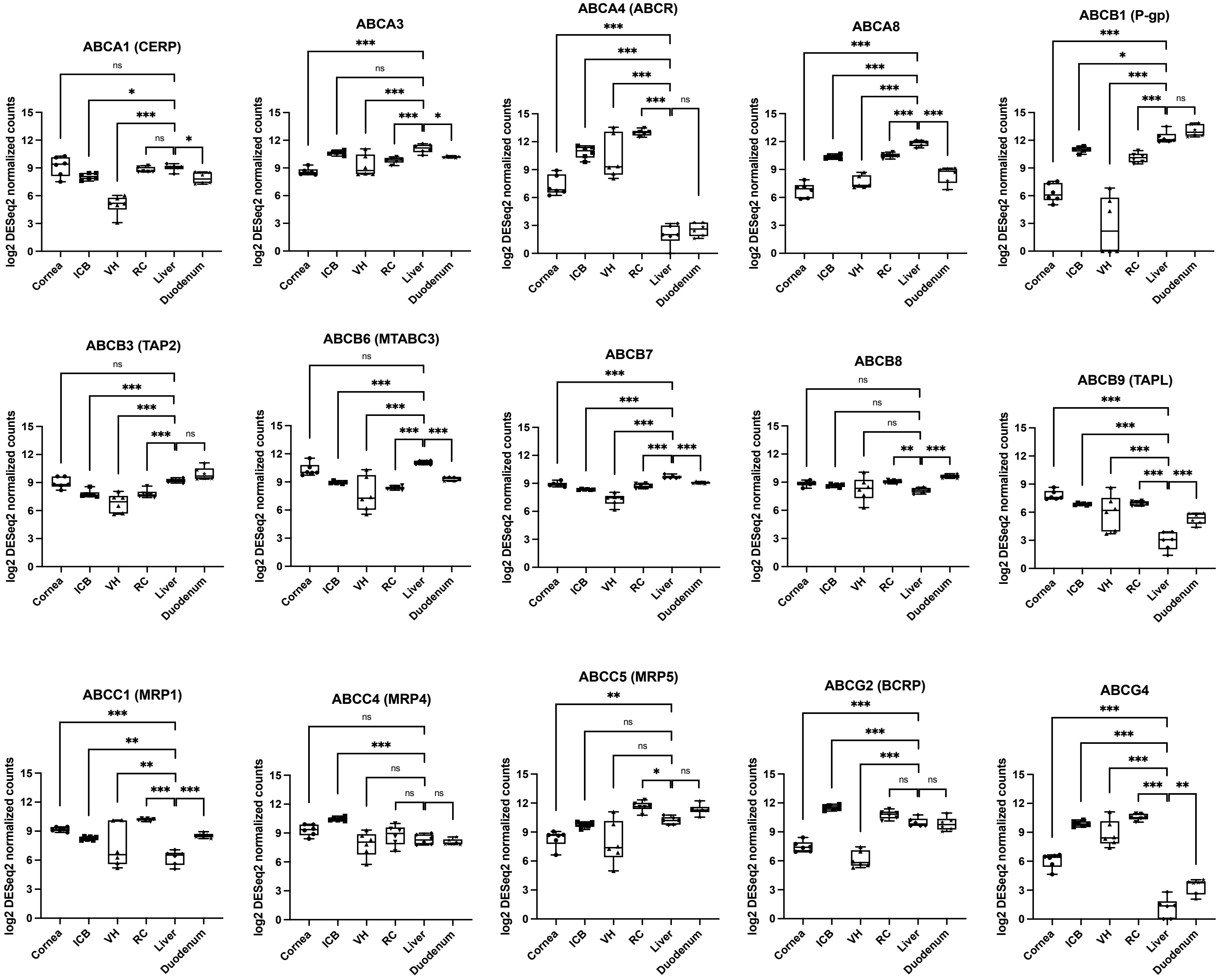

Supplemental Figure 15. The relative expression levels of ocular ABC transporters in the rabbit cornea, ICB, VH, RC, liver, and duodenum. The $\log_2$-transformed DESeq2 normalized counts were used to generate box and whiskers plots, and the B-H adjusted P-values from DESeq2 were employed to compare the gene expression levels in the ocular sub-tissues and duodenum to the liver, as described in the Materials and Methods.

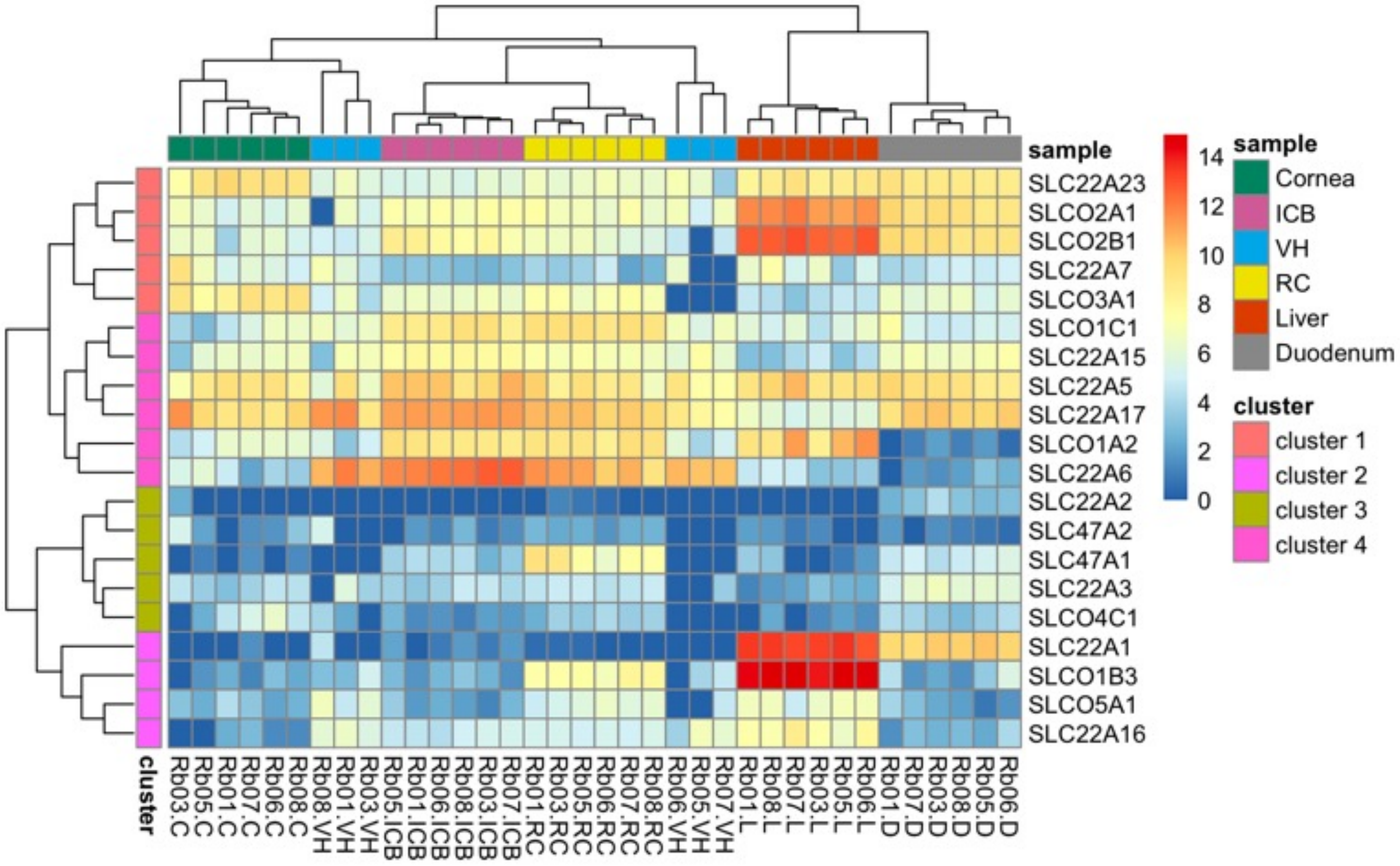

Supplemental Figure 16. The mRNA gene expression heatmap of major SLC transporters (n=20) in rabbit ocular sub-tissues, liver, and duodenum. The log2-transformed DESeq2 normalized counts were used to generate the heatmap and results were clustered as described in the Materials and Methods.

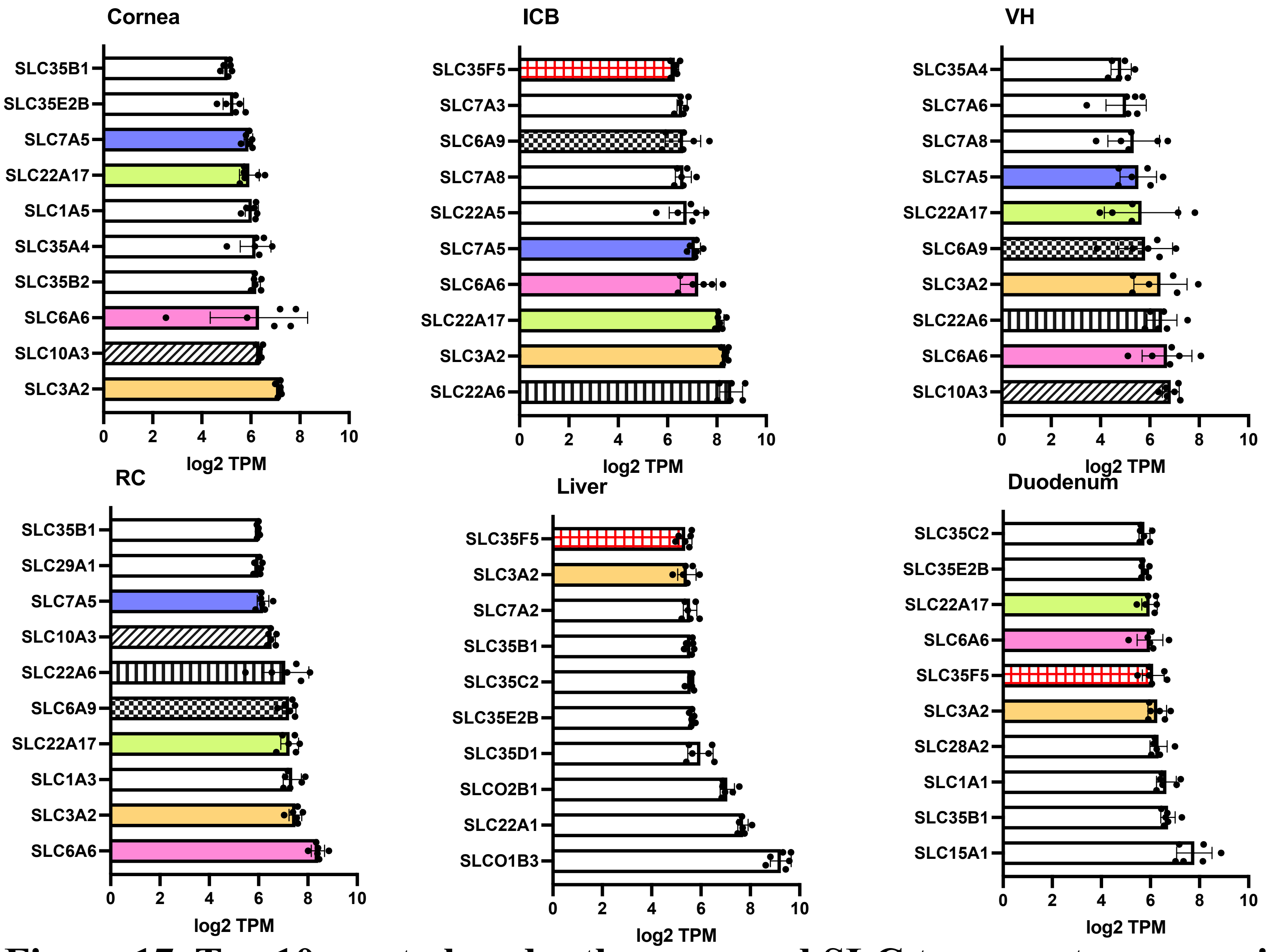

Supplemental Figure 17. Top 10 most abundantly expressed SLC transporters genes in the rabbit cornea, ICB, VH, RC, liver, and duodenum. The log2-transformed transcripts per million (TPM) values were used to rank the expression within each tissue type. Genes identified in the top-10 lists of multiple tissue types were color/pattern-coded to facilitate visual identification.

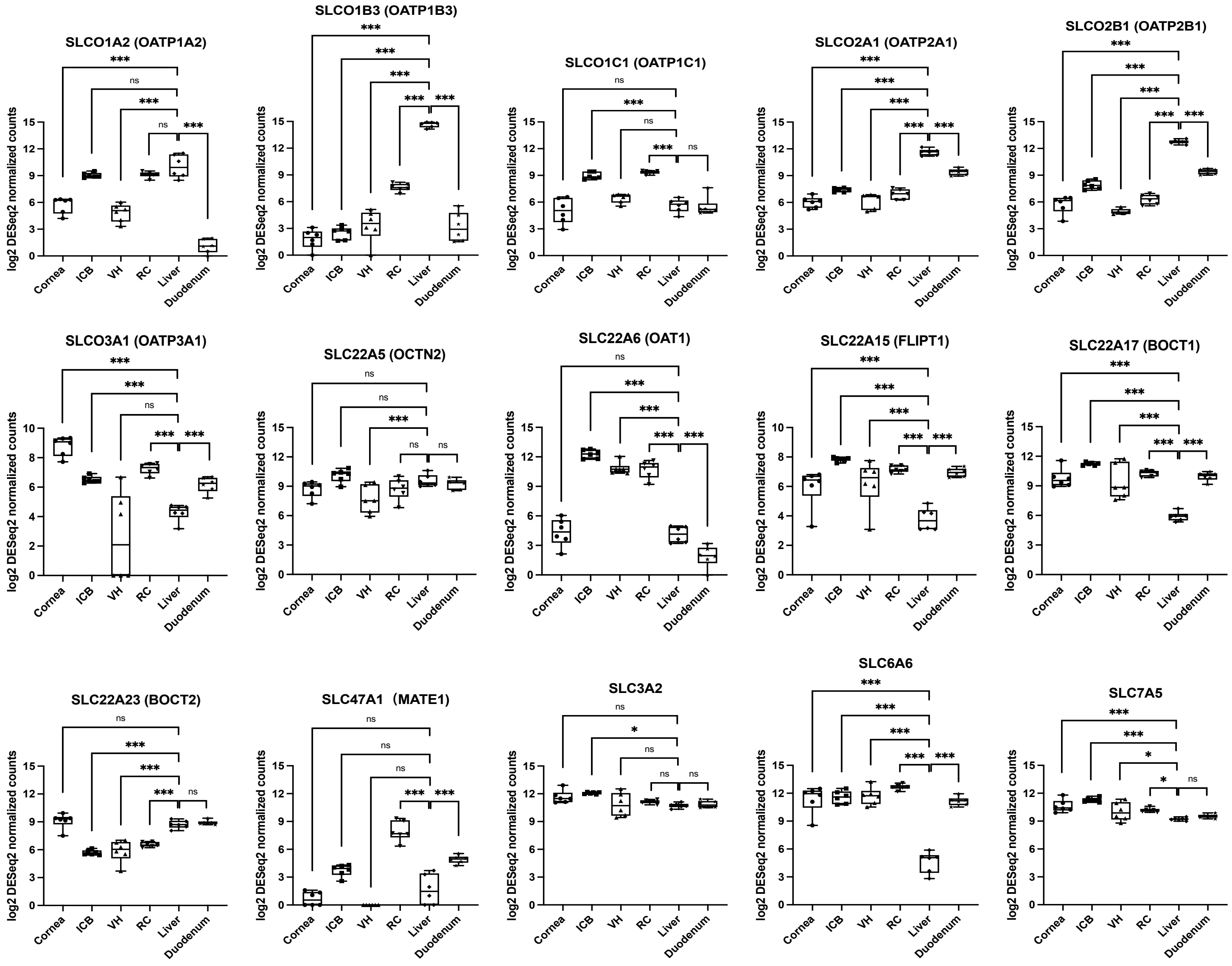

Supplemental Figure 18. The relative expression levels of ocular SLC transporters in the rabbit cornea, ICB, VH, RC, liver, and duodenum. The $\log_2$-transformed DESeq2 normalized counts were used to generate box and whiskers plots, and the B-H adjusted P-values from DESeq2 were employed to compare the gene expression levels in the ocular sub-tissues and duodenum to the liver, as described in the Materials and Methods.

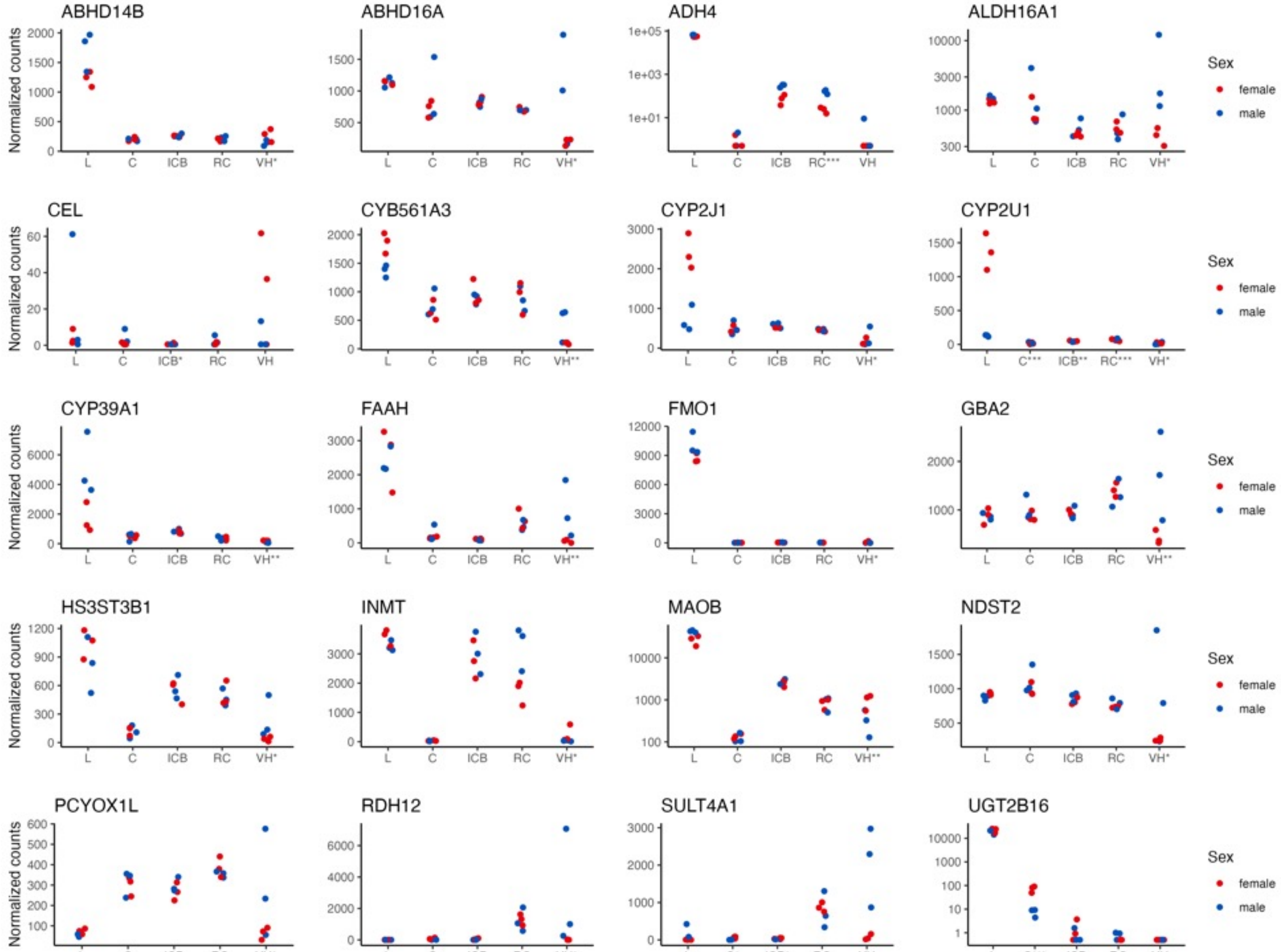

Supplemental Figure 19. Normalized counts from female (red) and male (blue) rabbits for DME genes with significant sex-by-tissue interactions. Asterisks next to tissue axis labels reflect FDR-adjusted P-values of sex-by-tissue interaction (* < 0.01, ** < 0.001, *** < 0.0001).

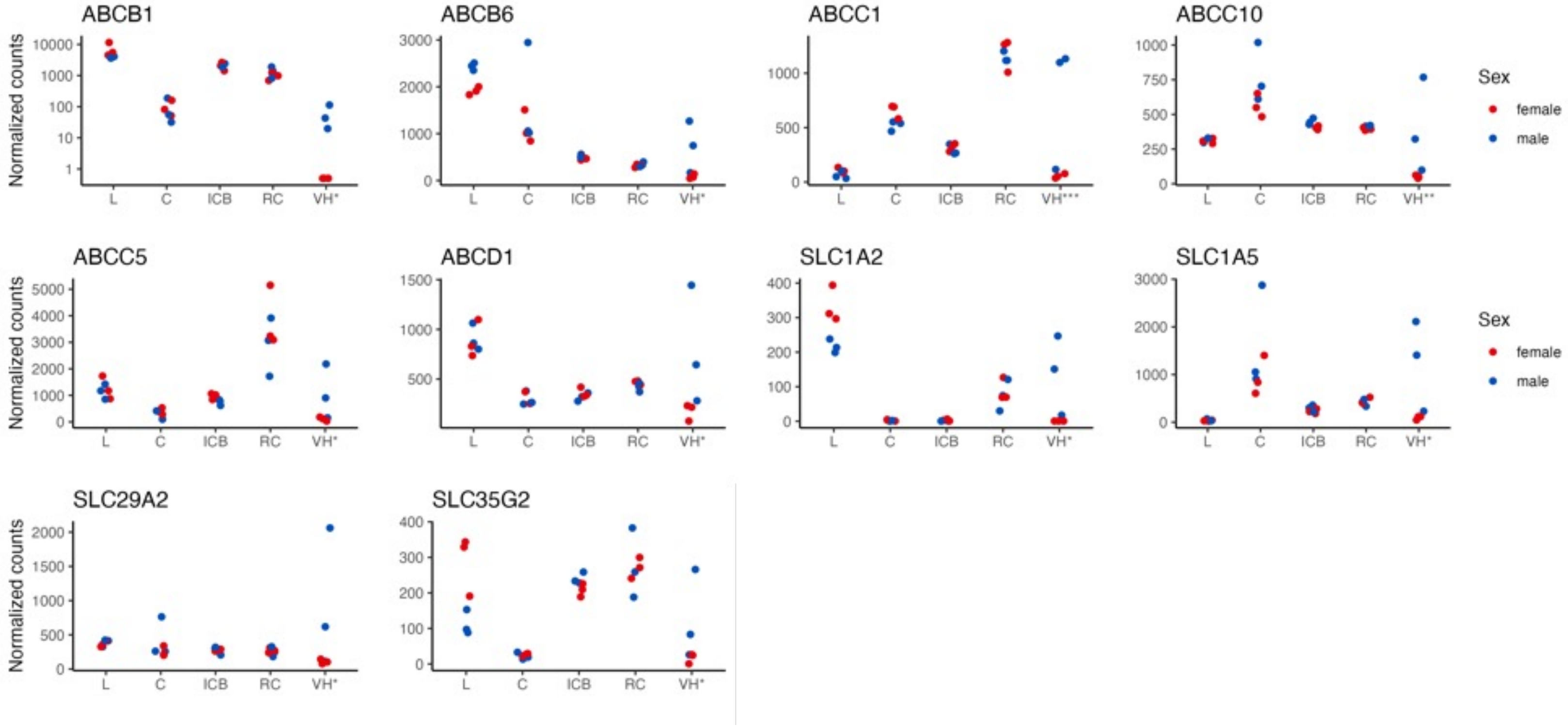

Supplemental Figure 20. Normalized counts from female (red) and male (blue) rabbits for DT genes with significant sex-by-tissue interactions. Asterisks next to tissue axis labels reflect FDR-adjusted P-values of sex-by-tissue interaction (* < 0.01, ** < 0.001, *** < 0.0001).
